# Gene duplications in the *E. coli* genome: common themes among pathotypes

**DOI:** 10.1186/s12864-019-5683-4

**Published:** 2019-04-24

**Authors:** Manuel Bernabeu, José Francisco Sánchez-Herrero, Pol Huedo, Alejandro Prieto, Mário Hüttener, Julio Rozas, Antonio Juárez

**Affiliations:** 1 Department of Genetics, Microbiology and Statistics, University of Barcelona, Barcelona, Spain; 2 Biodiversity Research Institute (IRBio), University of Barcelona, Barcelona, Spain; 3 Institute of Biotechnology and Biomedicine (IBB), Universitat Autònoma de Barcelona, Cerdanyola del Vallès, Spain; 4 Institute for Bioengineering of Catalonia, The Barcelona Institute of Science and Technology, Barcelona, Spain

**Keywords:** Pathotypes, Gene duplication, *Escherichia coli* 042, H-NS, Hha

## Abstract

**Background:**

Gene duplication underlies a significant proportion of gene functional diversity and genome complexity in both eukaryotes and prokaryotes. Although several reports in the literature described the duplication of specific genes in *E. coli*, a detailed analysis of the extent of gene duplications in this microorganism is needed.

**Results:**

The genomes of the *E. coli* enteroaggregative strain 042 and other pathogenic strains contain duplications of the gene that codes for the global regulator Hha. To determine whether the presence of additional copies of the *hha* gene correlates with the presence of other genes, we performed a comparative genomic analysis between *E. coli* strains with and without *hha* duplications. The results showed that strains harboring additional copies of the *hha* gene also encode the *yeeR irmA* (*aec69*) gene cluster, which, in turn, is also duplicated in strain 042 and several other strains. The identification of these duplications prompted us to obtain a global map of gene duplications, first in strain 042 and later in other *E. coli* genomes.

Duplications in the genomes of the enteroaggregative strain 042, the uropathogenic strain CFT073 and the enterohemorrhagic strain O145:H28 have been identified by a BLASTp protein similarity search. This algorithm was also used to evaluate the distribution of the identified duplicates among the genomes of a set of 28 representative *E. coli* strains. Despite the high genomic diversity of *E. coli* strains, we identified several duplicates in the genomes of almost all studied pathogenic strains. Most duplicated genes have no known function. Transcriptomic analysis also showed that most of these duplications are regulated by the H-NS/Hha proteins.

**Conclusions:**

Several duplicated genes are widely distributed among pathogenic *E. coli* strains. In addition, some duplicated genes are present only in specific pathotypes, and others are strain specific. This gene duplication analysis shows novel relationships between *E. coli* pathotypes and suggests that newly identified genes that are duplicated in a high percentage of pathogenic *E. coli* isolates may play a role in virulence. Our study also shows a relationship between the duplication of genes encoding regulators and genes encoding their targets.

**Electronic supplementary material:**

The online version of this article (10.1186/s12864-019-5683-4) contains supplementary material, which is available to authorized users.

## Background

Pathogenic *Escherichia coli* strains can cause either intestinal infections (which are diarrheagenic) or extraintestinal infections. Based on the type of virulence factors displayed and the strategy used to cause infection, *E. coli* strains are grouped into pathotypes. Some pathotypes are associated with diarrhea: enteropathogenic (EPEC), enterotoxigenic (ETEC), enterohemorrhagic (EHEC), enteroaggregative (EAEC) and enteroinvasive (EIEC) strains are the best characterized. Other pathotypes are common causes of urinary tract infections (uropathogenic *E. coli,* UPEC), newborn meningitis (neonatal meningitis *E. coli,* NMEC) or sepsis (SEPEC).

As mentioned above, enteroaggregative *Escherichia coli* (EAEC) strains are one of the groups of diarrheal *E. coli* pathogens [1]. EAEC strains can be distinguished from EPEC strains because of their different patterns of adherence to HEp-2 cells. Whereas EPEC strains display a “microcolony” pattern of adherence, EAEC strains display a characteristic aggregative or “stacked-brick” pattern [2]. EAEC adherence to intestinal cells is mediated by a specific type of fimbrial adhesin termed aggregative adherence fimbriae (AAFs). Epidemiological studies have shown that EAEC strains are genetically heterogeneous. A large number of virulence factors have been identified in EAEC clinical isolates [3]. Most of these virulence factors are expressed by only a percentage of all EAEC strains characterized. The strain O104:H4 is an example of EAEC genetic heterogeneity. A few years ago in Germany, this strain caused a large outbreak of bloody diarrhea [4]. Isolates from the O104:H4 outbreak harbor a plasmid (pAA) that encodes, among other virulence factors, the fimbriae that mediate the EAEC type of adherence [5]. Unlike typical EAEC strains, strain O104:H4 contains a prophage encoding the Shiga toxin [6], which is a well-characterized virulence determinant usually expressed by a different *E. coli* pathotype, EHEC [7].

Strain 042 is the best-characterized EAEC strain. It caused diarrhea in a volunteer trial [8]. The genome sequence of this strain is available [9], and its virulence factors are characterized. Strain 042 harbors the IncFIC virulence plasmid pAA2 [9, 10], which encodes the fimbrial adhesion determinants (the AAF/II variant of AAF), the virulence master regulator AggR and other virulence determinants [9, 11–13].

When analyzing the 042 genomic sequence, we noticed that unlike other *E. coli* strains, the chromosome of this strain encodes four paralogues of the *hha* gene: *hha, ydgT* and the novel *hha2* and *hha3* genes [14]. The *hha* gene product, the Hha protein, is representative of a family that includes a group of sequence-related low molecular mass proteins (approximately 8 kDa) involved in gene regulation in enterobacteria. These proteins interact with the nucleoid-associated protein H-NS to modulate gene expression (as reviewed in [15]). The genomes of several enterobacterial isolates, such as *Salmonella* and *E. coli* strains, encode a paralogue of the *hha* gene (the *ydgT* gene). Orthologues of *hha* are also present in several conjugative plasmids [16, 17]. The presence of the novel chromosomal *hha* paralogues *hha2* and *hha3* has been associated with pathogenic *E. coli* strains that belong to a wide range of pathotypes [14].

Gene duplication underlies a significant proportion of gene functional diversity and genome complexity [18–22]. Gene duplications occur in both eukaryotes and prokaryotes and significantly impact their gene repertoires [18–23]. In this work, we first aimed to gain insight into the biological role of the novel *hha2* and *hha3* genes of strain 042. To this end, we first performed a comparative genomic analysis between strains with and without *hha2/hha3*. This approach allowed us to correlate *hha2/hha3* with a gene cluster (the *flu yeeR* gene cluster), which is also duplicated in strain 042. Because strain 042 exhibits the duplication of genes encoding both regulators and the genes likely targeted by regulators, we decided to determine the extent of gene duplications in this strain and in the genomes of other pathogenic *E. coli* strains. Our analysis uncovers interesting patterns of gene duplications that are common to strains belonging to several *E. coli* pathotypes, both diarrheagenic and nondiarrheagenic.

## Methods

To investigate the pan-, core, variable, and exclusive genomes of *E. coli hha*^+^ (*hha2/3*^+^) and *hha*^−^ strains, two sets of five representative strains were considered. The *E. coli* strains in the *hha2/3*^+^ set were 042, NA114, O104:H4 LB226692, ETEC H10407 and UMN026. The *E. coli* strains in the *hha*^*−*^set were O111:H-11128, 53,638, IAI39, O127:H6 E2348/69 and O157:H7 Sakai (see Additional file 1: Table S1 for details).

Genomic analyses were performed using the MaGe Pan/Core genome tool (http://www.genoscope.cns.fr/agc/microscope/compgenomics/pancoreTool.php), and protein families were determined using MicroScope gene families (MICFAM) [24] with the following parameters: 80% amino acid identity and 80% alignment coverage.

For the identification of putative duplicates, we retrieved and downloaded the translated coding sequences of 28 *E. coli* strains from GenBank (Additional file 1: Table S1). For the BLAST search analysis, we used as filtering parameters a similarity cutoff > 85%, an alignment length between pairs > 85% and an *e*-value < 10^− 10^.

We analyzed the extent of gene duplication among strains by performing an all-vs-all BLASTp [25] protein similarity search (i.e., with the translated coding sequence regions of each strain, filtering the results according to the parameters specified above). For each duplicate, we retrieved genomic features (from the GenBank genomic feature files-gff), plotted the coordinates using R [26] and colored the duplicates according to their groups.

For the gene duplication analysis between strains and for the identification of the presence/absence of putative duplicated encoded proteins/coding regions, we also employed BLASTp. We searched the putative duplicates of interest against all translated coding sequences (all six frames). The results were filtered according to the above cutoff parameters.

In silico operon prediction was performed using the FGENESB program (Softberry, Inc., Mount Kisco, NY) (http://www.softberry.com/).

The bioinformatics scripts employed for the analysis were deposited and available at the github website: https://github.com/molevol-ub/BacterialDuplicates.

Statistical analysis. Proportions were compared between groups by using the two-tailed Fisher’s exact test. A *P*-value of less than 0.05 was considered significant.

For the RNA-seq experiments, the detailed information and raw data were previously published in [27].

## Results

### *E. coli* strains encoding *hha2/hha3* usually encode the *flu yeeR aec69 aec70* cluster, which is also duplicated

To gain insight into the biological role of *hha* duplication in the EAEC strain 042, we hypothesized that the presence of multiple alleles of a global regulator could be associated with the presence of genes specifically targeted by the regulator. To support this hypothesis, we decided to compare the core genomes of two groups, each with five *E. coli* strains. One of them included representatives that encode *hha2/hha3* (*hha*^*+*^), and the other included strains that do not encode them (*hha*^*−*^). To identify those genes that are truly exclusive to the *hha*^*+*^ set, we used a restrictive strategy of excluding the pangenome of the *hha*^*−*^ set from the core genome of the *hha*^*+*^ set. By using this approach, only three gene families could be identified in the *hha*^*+*^ set: the *hha*, *yeeR* and *aec69* genes (Additional file 1: Figure S1 and Additional file 2).

**Fig. 1:**
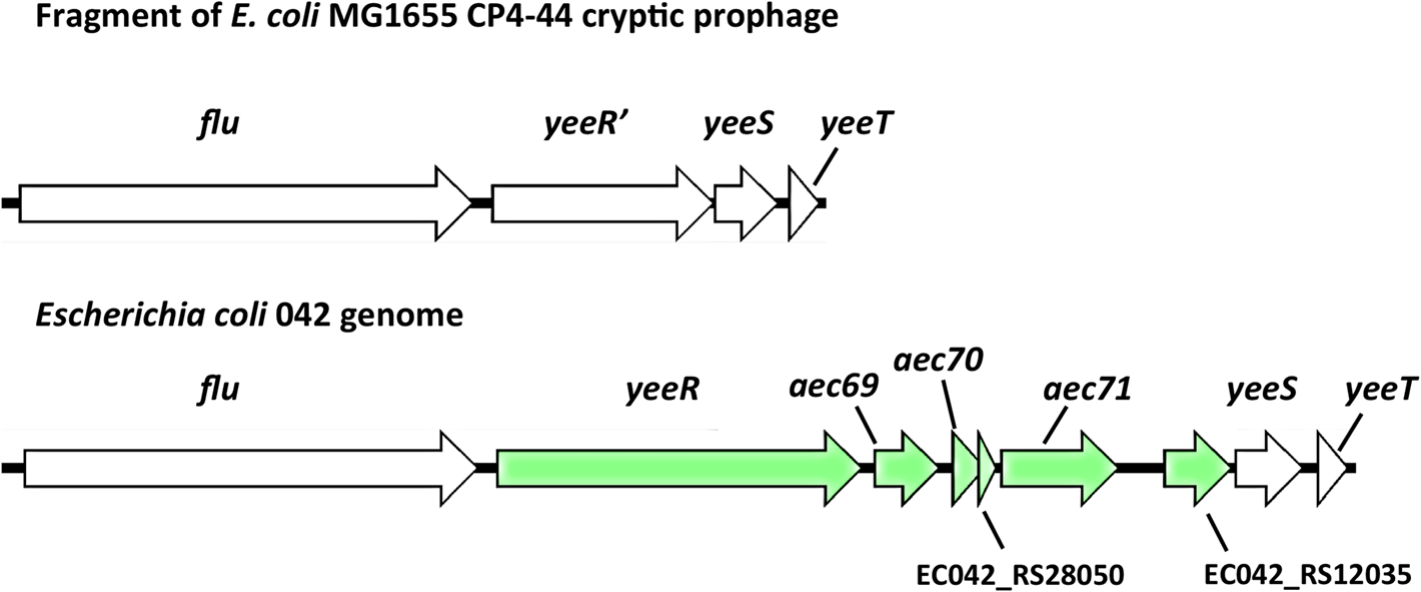
*flu yeeR* gene cluster in *E. coli*. Schematic representation of the genetic cluster comprising the *flu, yeeR, yeeS and yeeT* genes in *E. coli* MG1655 prophage CP4–44 and *flu, yeeR, irmA* (*aec69*)*, aec70, aec71, yeeS and yeeT* in *E. coli* 042

**Fig. 2:**
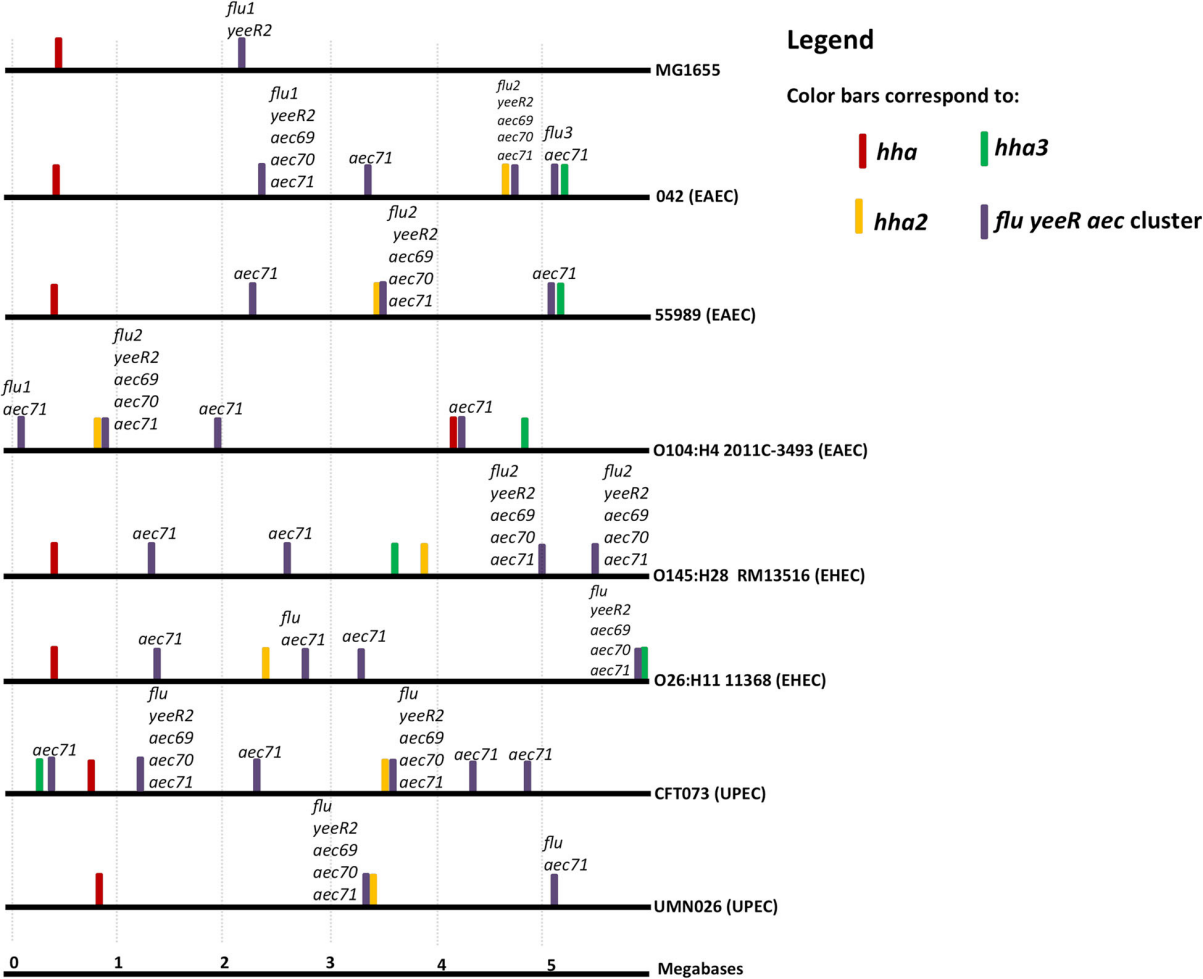
Map position of the *hha* (red bar), *hha2* (orange bar), *hha3* (green bar) and genes from the *flu yeeR aec* gene cluster (violet bar) in the chromosomes of *E. coli* MG1655 and seven other *E. coli* strains belonging to different pathotypes (as indicated in each case). Note the duplication of genes belonging to the *flu yeeR aec* gene cluster in several strains. Scale bars 0 to 5 correspond to megabases in the genetic map

*The yeeR* and *aec69* genes belong to a gene cluster that includes *flu* (whose gene product is the well-characterized antigen43 protein), *aec70* and *aec71* (Fig. 1). A recent report shows that *aec69*, termed *irmA*, is transcribed in a single transcriptional unit with *flu* and *yeeR* [28]. In *E. coli* K12, the *yeeR* gene is truncated, and the *irmA* (*aec69*)*, aec70* and *aec71* genes are missing (Fig. 1)*.* This cluster belongs to the prophage CP4–44. Taking into account the high genomic variability of *E. coli,* the identification of *yeeR* and *irmA* (*aec69)* as linked to *hha2/hha3* when the two five-strain groups were compared does not exclude the possibility that other strains that do not encode *hha2/hha3* might encode *yeeR/irmA* (*aec69*) or that other strains harboring *hha2/hha3* do not harbor *yeeR/irmA* (*aec69*). To improve the analysis, we performed a BLASTp search on a total of 28 *E. coli* genomes, including both commensal and pathogenic strains belonging to several pathotypes (Additional file 1: Table S1). The results obtained showed that 72% of the strains expressing *hha2/hha3* also express the *yeeR* or *irmA* (*aec69*) genes (*P*-value < 0.05), while 61% also express *flu* or *aec70* (this latter comparison was close to the critical value (*P* = 0.055)) (Additional file 1: Table S2). In contrast, only 20–40% of *hha2/hha3*^*−*^ strains express *yeeR, irmA (aec69), flu* and *aec70*. *aec71* does not appear to be associated with *hha2/hha3*. Its presence is widespread in both *hha2/hha3* and *hha2/hha3*^*−*^ strains. We then analyzed the map positions of the *hha2/hha3* genes and the *flu yeeR irmA* (*aec69*) *aec70 aec71* gene cluster in the chromosomes of seven *E. coli* strains corresponding to different pathotypes, including both enteric and extraintestinal pathogens (Fig. 2). In several instances, *hha2/hha3* mapped close to the *yeeR irmA* (*aec69*) *aec70 aec71* gene cluster. This study also showed that in most of the virulent *E. coli* strains analyzed (including the EAEC strain 042), genes belonging to the *yeeR irmA* (*aec69*) *aec70 aec71* cluster are also duplicated (Fig. 2). The presence in the chromosome of strain 042 of four copies of *hha-*like genes (*hha, ydgT, hha2* and *hha3* [14]), three copies of *hns*-like genes (*hns, stpA* and *hns2*) [27], two copies of *yeeR* and *irmA* (*aec69*), three copies of *flu* and four copies of the *aec71* gene suggests that gene duplication may play a relevant role in this and perhaps other pathogenic *E. coli* strains. We therefore decided to investigate the extent of gene duplications first in the genome of strain 042 and thereafter in the genomes of other pathogenic *E. coli* strains.

### Gene duplications in the EAEC strain 042 genome

**Fig. 3:**
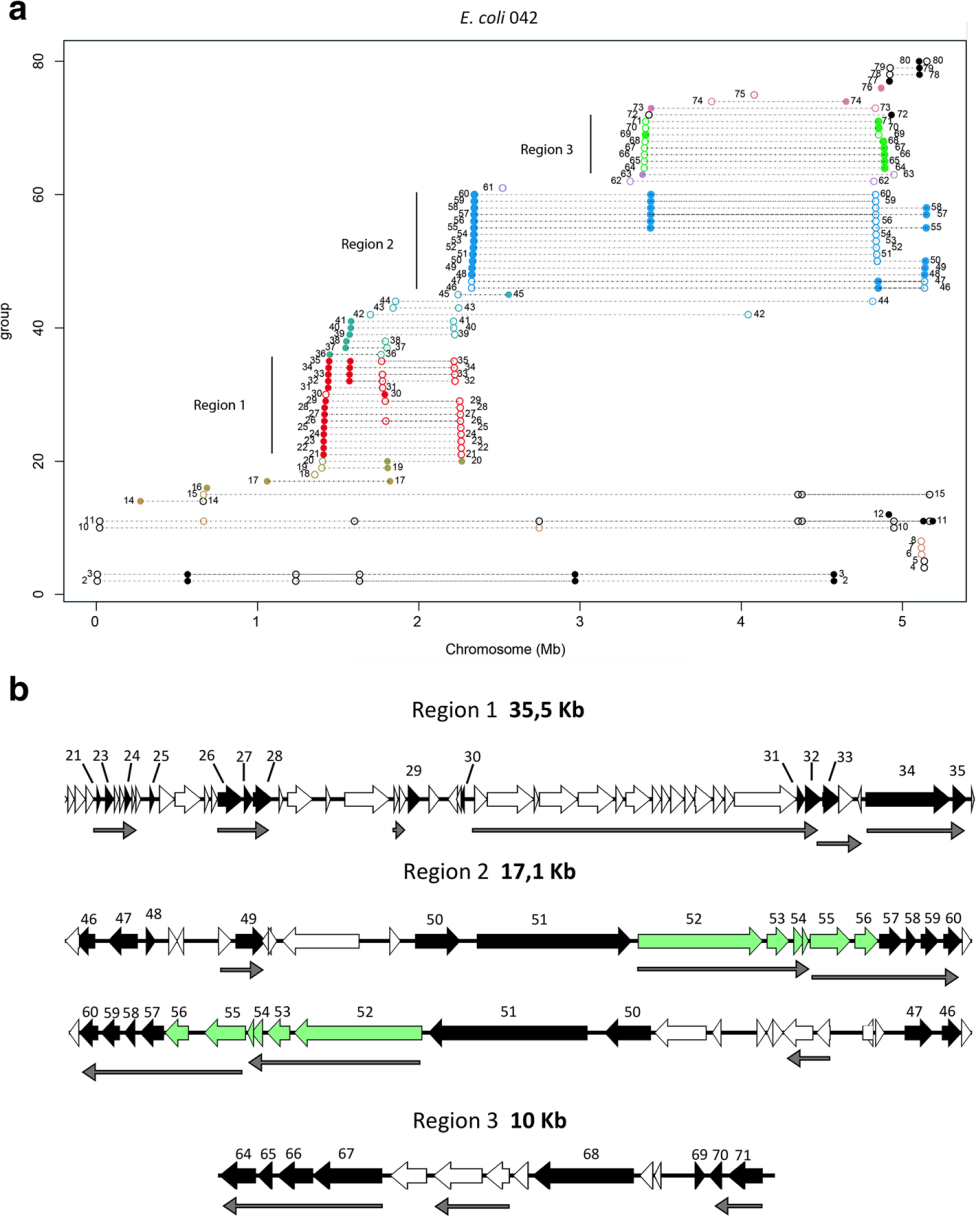
**a** Genes duplicated in the *E. coli* strain 042. The X axis corresponds to the linear map of the chromosome. Each group of spots connected by a horizontal dashed line corresponds to a single gene duplicated in different positions on the chromosome. The different spots indicate the map positions of the different copies of the gene. Point shapes represent the strand on which a protein is encoded: filled circle for (+) strand and circle for (−) strand. Numbers correspond to the different duplicated genes, which have been numbered by their order starting from the origin of the chromosomal map. Genes numbered 2 to 5, 10 to 12, 77 to 80 (black closed and open circles) correspond to transposases. Colors and vertical bars define the three main regions that contain duplicated genes. Duplications 1, 9 and 13 are not shown because both repeated copies map to the pAA plasmid (not shown in the figure). Duplications 4 to 8 contain one copy in the chromosome (shown) and the other in the plasmid (not shown). **b** Details of regions 1 to 3, showing duplicated genes (labeled in black). To show inversion, both copies of region 2 are shown. Genes labeled in green correspond to the *flu yeeR* gene cluster. Thin gray arrows correspond to the in silico operon prediction. The figure was generated using Easyfig [40]. See Additional file 1: Table S3 for the function of each duplicated gene

We analyzed the extent of gene duplications in strain 042 by using the BLASTp algorithm (see the materials for details) and mapped along the 042 genome those genes that are present in two or more copies (Fig. 3a). A total of 80 genes were duplicated in strain 042. Some of these genes correspond to transposases (black and open circles). Most of the duplicated genes are clustered in three main regions (labeled with vertical bars), which we arbitrarily termed regions 1 to 3. A significant number of genes that map to region 1, which is approximately 35.5 kb long, correspond to phage genes (Fig. 3b, Additional file 1: Table S3). Region 2 is approximately 17 kb long and contains the *flu yeeR irmA* (*aec69*) *aec70 aec71* cluster, a toxin-antitoxin gene and several other genes of unknown function (Fig. 3b, Additional file 1: Table S3). One of the copies of this region includes the *hha2* gene. The two copies of region 2 are inverted in the 042 chromosome, suggesting that genetic rearrangements leading to gene duplication can affect this region as a single recombinational unit. Region 3 is 10 kb long and includes mostly genes of unknown function (Fig. 3b, Additional file 1: Table S3).

We also analyzed gene duplications in strain 042 by using a BLASTn algorithm, yielding results similar to those obtained by using BLASTp (Additional file 1: Figure S2).

### Duplicated genes in regions 1 and 2 are repressed by the H-NS/Hha system

Considering that some duplicated genes of region 2 in strain 042 (i.e., the *yeeR irmA* (*aec69*) gene cluster) have been identified as linked to *hha2/hha3*, it can be hypothesized that some duplicated genes are regulated by the H-NS/Hha system. To support this hypothesis, we analyzed the previously reported transcriptional profiles of strain 042 and its *hha* null (*hha hha2*) and *hns* mutant derivatives [27], which was performed in cultures growing in LB medium at 37 °C. We assessed whether the duplicated genes of the three regions of strain 042 show H-NS- or Hha-dependent regulation (Table 1). All genes from region 2 show fold change values higher than 2, both in the *hha* null and *hns* derivatives. This result was also observed for several genes in region 1. Only two genes from region 3 appear to be coregulated by H-NS/Hha.

**Table 1:** Comparative expression of the duplicated genes from regions 1 to 3 of strain 042 in the *hha* null (deletion of the *hha* and *hha2* alleles) and *hns* mutants. Values indicate the fold change with respect to the wt strain. Fold change values higher than two are considered significant

	group	locus tag	*hha null*	*hns*
Region 1	21	EC042_1328	2.1	6
	23	EC042_1330	2.6	5
	24	EC042_1333	2.5	4.2
	25	EC042_1336	1.8	0.6
	26	EC042_1342	3.5	4.8
	27	EC042_1343	2.8	4.8
	28	EC042_1344	1.7	4
	29	EC042_1349	2.4	3.8
	30	EC042_1353	4.3	3.3
	31	EC042_1371	1.5	6.1
	32	EC042_1372	1.5	3.8
	33	EC042_1373	1.6	3.3
	34	EC042_1376	2	3.7
	35	EC042_1377	2.4	4.2
Region 2	46	EC042_2236A	2.9	2.9
	47	EC042_2237	3.8	4.5
	48	EC042_2238	5.6	4.2
	49	EC042_2239	3.8	3
	50	EC042_2241	6.2	4.8
	51	EC042_2242	3.1	3.9
	52	EC042_2243	3	3.8
	53	EC042_2244	2.2	2.2
	54	EC042_2244A	4.9	5.9
	55	EC042_2245	5	5.1
	56	EC042_2246	4.6	4.8
	57	EC042_2247	5	3.9
	58	EC042_2247A	5	4.7
	59	EC042_2248	5.1	4.5
	60	EC042_2249	4.9	2.6
Region 3	64	EC042_3180	3	4.4
	65	EC042_3181	3.4	2.4
	66	EC042_3182	2.1	2.2
	67	EC042_3183	1.9	1.3
	68	EC042_3187	3.8	1.1
	70	EC042_3190	0.6	0.3
	71	EC042_3191	1.9	1.6

### Genes from 042 regions 1 and 2 are also duplicated in several other pathogenic *E. coli* strains

**Fig. 4:**
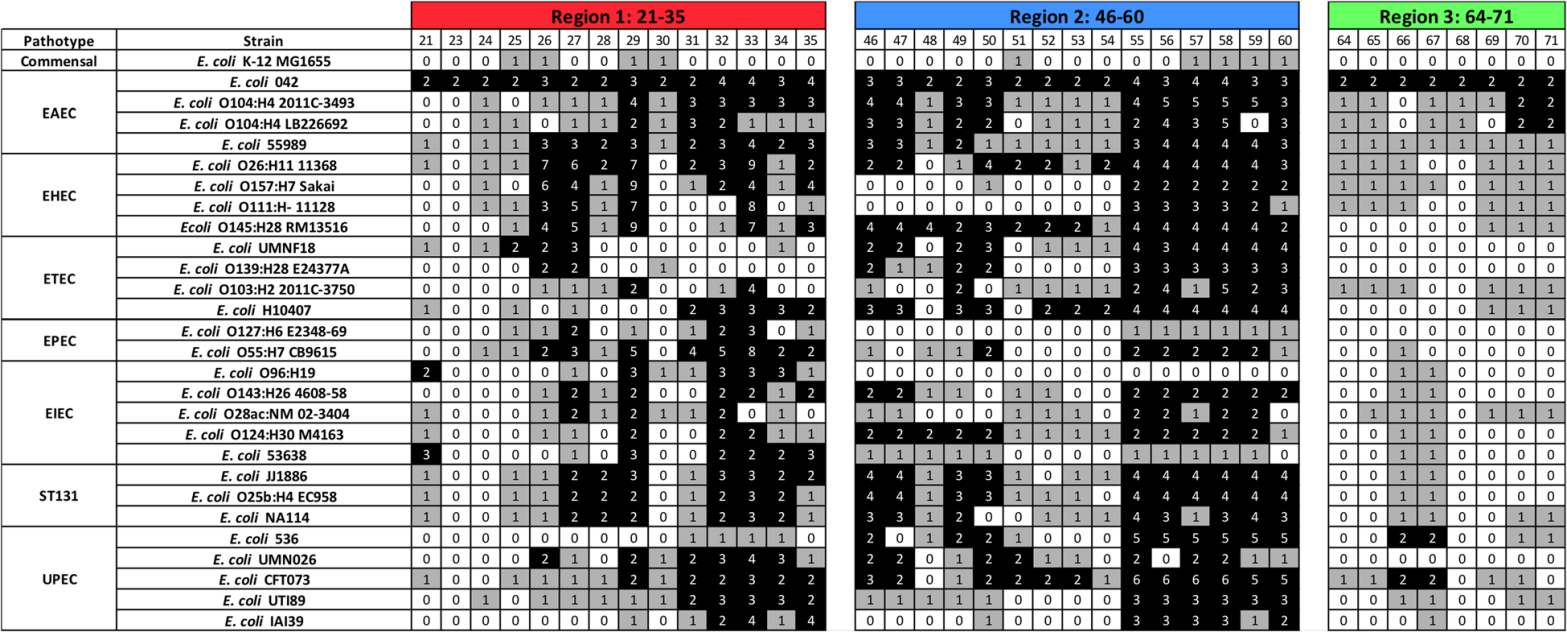
Distribution of the detected duplicated genes in other *E. coli* strains belonging to a wide range of pathotypes. BLASTp analysis was used for the study. White color, gene absent. Gray color, gene present in a single copy. Black color, gene present in two or more copies. The numbers show the copy number of each gene. Note that a significant number of the duplicated genes are absent in strain MG1655

After determining the extent of gene duplications in the genome of strain 042, we addressed the question of whether the existing duplicates in strain 042 were strain-specific or whether they were generated in some putative ancestor and are also present in many other *E. coli* strains. We used the DNA sequences of the selected 28 *E. coli* genomes to perform a gene duplication analysis (see the methods for details) and annotated the number of copies of each of the duplicated genes from strain 042 that were detected in each of the other genomes (Fig. 4). With respect to region 1, 10 out of the 14 duplicated genes in strain 042 were duplicated in most of the genomes analyzed. With respect to region 2, the 15 duplicated genes are duplicated in either some or most of the genomes studied (Fig. 4). Six of the genes from that region (listed as 55 to 60), which appear as a single linkage group and belong to the same putative transcriptional unit, are present in several copies (4 to 6) in the genomes of most of the strains. These genes encode conserved hypothetical proteins (55, 58–60), a putative antirestriction protein (56) and a putative DNA repair protein (57). With regard to region 3, duplications of the eight genes identified in strain 042 are a specific feature of that strain. Several of these genes are either absent or present in a single copy in most of the genomes studied (Fig. 4). It is relevant to mention here that only 9 out of 40 duplicated genes from strain 042 that map to regions 1 to 3 are present in a single copy in the genome of strain MG1655. The rest of the genes are not present in the genome of the commensal strain.

### Gene duplications in the genomes of strains CFT073 (UPEC) and O145:H28 (EHEC)

**Fig. 5:**
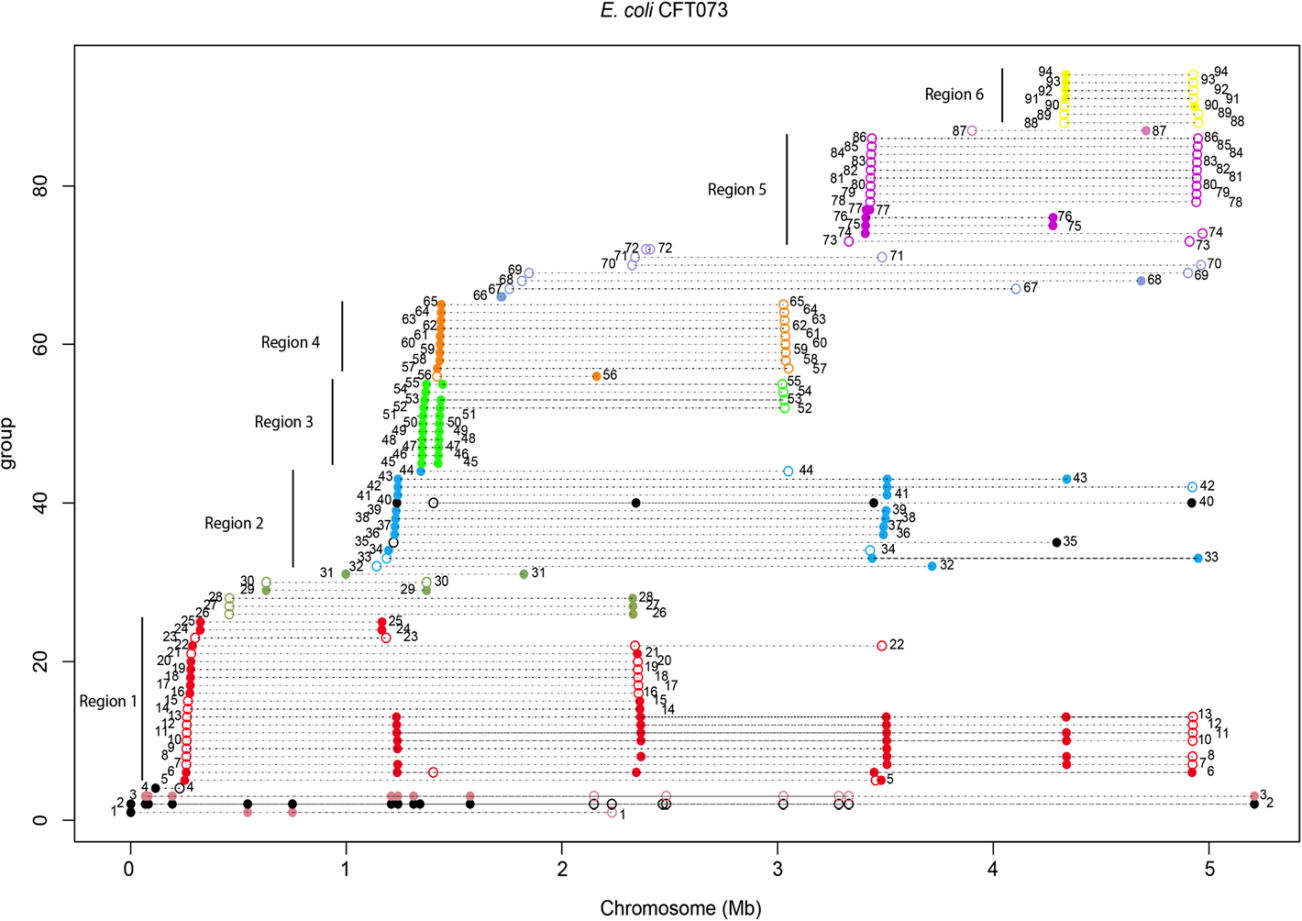
Genes duplicated in the *E. coli* strain CFT073. The X axis corresponds to the linear map of the chromosome. Each group of spots connected by a horizontal dotted line corresponds to a single gene, which is shown to be duplicated in different positions of the chromosome. The different spots indicate the map positions of the different copies of the gene. Point shapes represent the strand on which a protein is encoded: filled circle for (+) strand and circle for (−) strand. Numbers correspond to the different duplicated genes, which have been numbered by their order starting from the origin of the chromosomal map. Black closed and open circles correspond to transposases. Colors and vertical bars define the six main regions that contain duplicated genes. See Additional file 1: Table S4 for the function of each of the duplicated genes

To obtain a more complete picture of gene duplications in *E. coli*, we decided to analyze the genomes of two other *E. coli* strains that belong to pathotypes different from that of strain 042. Strain CFT073 is uropathogenic (UPEC), and strain O145:H28 is enterohemorrhagic (EHEC). With respect to strain CFT073, 94 duplicated genes could be identified. They can be grouped into six different DNA regions (Fig. 5, Additional file 1: Table S4). Some of these genes correspond to transposases, similar to strain 042.

**Fig. 6:**
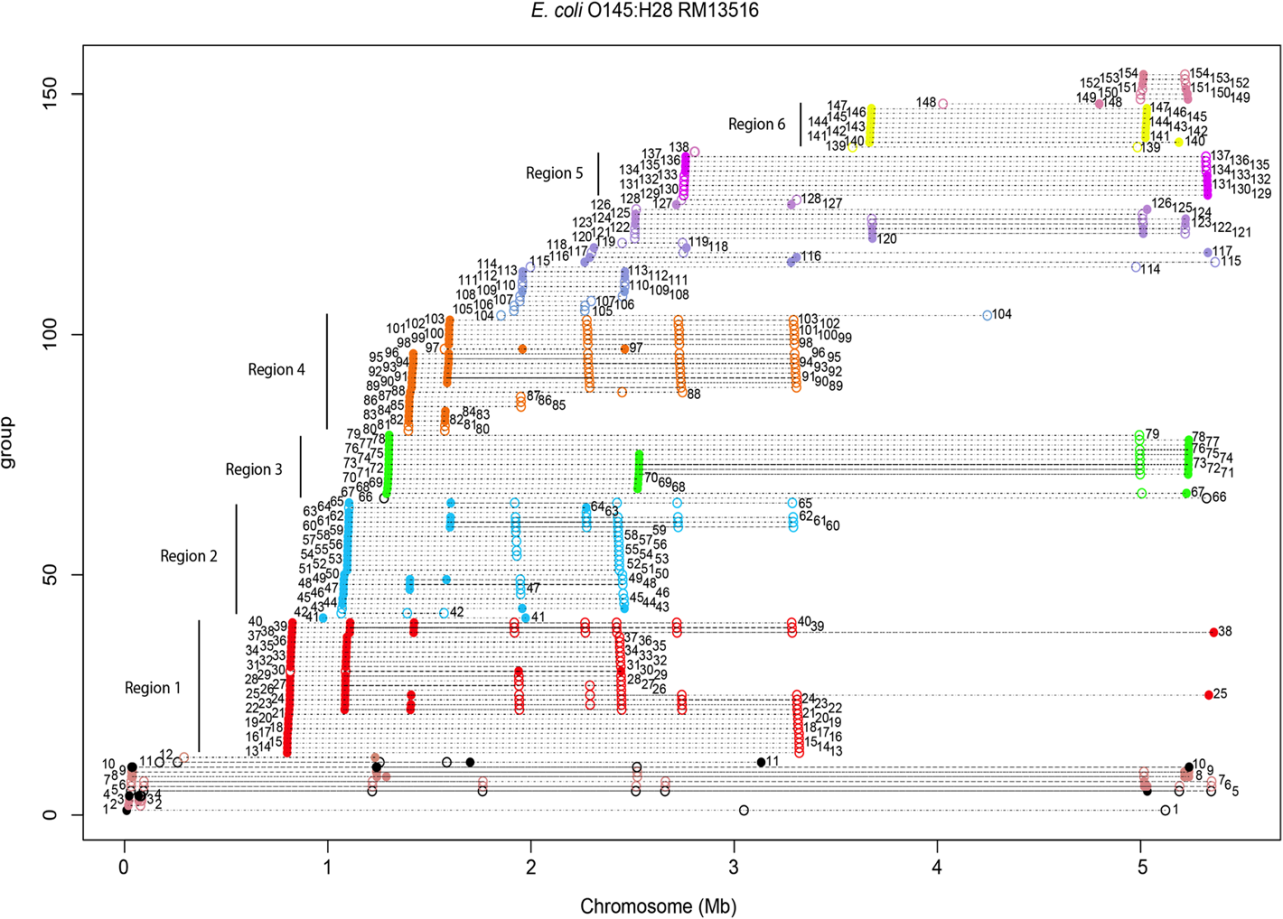
Genes duplicated in the *E. coli* strain O145:H28. The X axis corresponds to the linear map of the chromosome. Each group of spots connected by a horizontal dotted line corresponds to a single gene, which is shown to be duplicated in different positions of the chromosome. The different spots indicate the map positions of the different copies of the gene. Point shapes represent the strand on which a protein is encoded: filled circle for (+) strand and circle for (−) strand. Numbers correspond to the different duplicated genes, which have been numbered by their order starting from the origin of the chromosomal map. Black closed and open circles correspond to transposases. Colors and vertical bars define the six main regions that contain duplicated genes. See Additional file 1: Table S5 for the function of each duplicated gene

A total of 154 duplicated genes could be identified in the genome of strain O145:H28. The duplicated genes can also be grouped into six regions (Fig. 6, Additional file 1: Table S5). In this strain, several of the identified genes are present in more than two copies (Fig. 6). After identifying the duplicated genes in strains CFT073 and O145:H28, we also determined which of them are also duplicated in other *E. coli* strains. The genomic DNA sequences of the 28 *E. coli* strains were used to perform gene duplication analysis, and the number of copies of each of the duplicated genes from strains CFT073 and O145:H28 that were detected in each genome of the 28 *E. coli* strains was annotated. For both strains, duplicated genes that also occur as duplicates in other *E. coli* strains correspond to those already identified in strain 042 (Additional file 1: Figures S3 and S4). Some genes appear to be strain specific, as observed for strain 042. Some duplications in strains CFT073 and O145:H28 revealed a novel pattern: they are pathotype specific. The duplicated genes from strain CF703 region 5 belong to that group. Interestingly, most of these genes encode putative fimbrial proteins (Additional file 1: Table S4). Another example corresponds to duplications mapping in the region 4 of strain O145:H28. These genes are duplicated only in all the EHEC strains, one EPEC strain and one ETEC strain. Several of those genes are phage genes (Additional file 1: Table S5). In contrast to the EAEC strain 042, duplicated genes in the EHEC strain O145:H28 are not duplicated in the UPEC strains.

## Discussion

The existence of gene duplications in both eukaryotes and prokaryotes has been extensively studied [18–23, 29, 30]. Several reports have established the basis for how gene duplication and divergence generate families and superfamilies of proteins [21]. Gene duplications have been associated to the adaptation of cells to a changing environment [31, 32], and have been found to occur more frequently among HGT genes than among indigenous genes [33]. The presence of several copies of genes such as *flu* in some *E. coli* strains was previously reported [30, 32, 34–36].Nevertheless, detailed information about the extent of gene duplications in the genomes of the different types of pathogenic *E. coli* strains is needed. We applied an extensive blast search to identify putative internal duplications in the 042 strain using a moderate parameter cutoff (BLAST cutoff: > 85% similarity, > 85% alignment length and *e*-value < 10^− 10^) and found that most duplicates cluster together in specific regions of the 042 genome. The results obtained suggest that different mechanisms underlie these duplication events. Whereas the duplication of region 2 from strain 042 involves an inversion, this was not the case for regions 1 and 3. Interestingly, the genes in region 1 have a phage origin and are widespread in several strains. It is worth mentioning that, as a general rule, duplications result in the presence of copies of the duplicated gene in both strands of the *E. coli* chromosome. It is also remarkable that a significant number of the duplicated genes are organized in putative transcriptional units (Fig. 3b), thus suggesting the existence of coordinated expression in response to specific stimuli.

The comparative analysis of gene duplications in *E. coli* strains belonging to different pathotypes provides relevant information that can contribute to our understanding of the virulence mechanisms of this pathogen and better establish the relationships among the *E. coli* pathotypes. The existence of a significant number of genes that are duplicated in a wide range of pathotypes but absent from commensal strains suggests that these genes can play a relevant role in *E. coli* virulence. Genes 55 to 60 from strain 042 region 2 are duplicated in all except three of the 26 pathogenic *E. coli* strains analyzed. Given that detailed information about the function of the products encoded by a large number of these genes is missing, assigning functions to them and to many other genes of unknown function is a critical issue for better understanding the ability of *E. coli* to cause disease.

In addition to identifying a set of duplicated genes that is widespread in the different *E. coli* pathotypes, our study provides additional novel information on genomic features of virulent *E. coli* strains. In *E. coli,* some gene duplication processes are restricted to either specific strains or specific pathotypes. Examples are the duplicated genes in region 3 of strain 042 or the duplicated genes in regions 5 and 4 from the UPEC strain CFT073 and the EHEC strain 0145:H28, respectively. The study of the function of these genes can also contribute to a better understanding of the mechanisms underlying virulence in these pathotypes. It is well known that UPEC strains express specific types of fimbriae. Some of the duplicated genes in region 5 from the UPEC strain CFT073 encode putative fimbrial proteins, which might play a role in UPEC pathogenesis.

The correlation we observed between *hha* duplication and the presence of the duplicated *yeeR irmA* (*aec69*) gene cluster suggested that Hha (and H-NS) could modulate the expression of duplicated *E. coli* genes. The analysis of the comparative expression of duplicated genes in the wt 042 strain and its isogenic *hha* null and *hns* derivatives shows that under specific growth conditions (LB medium, 37 °C), H-NS/Hha proteins downregulate the expression of a significant number of duplicated genes. These data highlight a novel role for the H-NS/Hha proteins in silencing several of the genes that are duplicated in strain 042. Hence, it can be hypothesized that to avoid fitness costs, duplications of genes targeted by global regulators may require the duplication of the genes that encode them. Derepression of H-NS/Hha-silenced genes can occur when environmental conditions change. Then, gene duplication may be advantageous because the two copies can exhibit different expression patterns and/or respond to different stimuli. This is the case for the duplicated *irmA* gene in strain 042 (our unpublished results).

A relevant point is whether HGT processes are underlying the presence of gene duplications in strain 042. The duplicated genes that map in the region 1 of strain 042 are of phage origin and can hence be considered as HGT DNA. In any case, a detailed phylogenetic analysis is being undertaken now to assess the origin of all duplicates that map in the three regions identified in strain 042.

Finally, our study has also shown some novel relationships between *E. coli* pathotypes. It is remarkable that most of the duplicated genes in the EAEC strain 042 are also duplicated in UPEC strains. Previous studies have suggested a close relationship between EAEC and UPEC strains [33, 37]. In fact, *E. coli* strains showing a hybrid UPEC/EAEC genotype have been isolated [38]. The similar gene duplication patterns of EAEC and UPEC strains further support this EAEC/UPEC relationship. Unlike EAEC strain 042, duplicated genes in the EHEC strain O145:H28 are usually duplicated in EPEC and ETEC strains but not in UPEC strains. A distinctive feature of EHEC strains is that some of the duplicated genes are present in more than two copies.

For some *E. coli* infections, such as those caused by ETEC, the effectiveness of the existing vaccines must be significantly improved [39]. If any of the gene products encoded by the identified duplicated genes are antigenic, they could be candidates for developing novel improved *E. coli* vaccines.

## Conclusions

Duplications of the *hha* gene can be correlated with the presence of genes belonging to the *flu yeeR aec* gene cluster, which is also duplicated in several pathogenic *E. coli* strains. The analysis of gene duplications in the *E. coli* genome has shown that (i) a number of duplicated genes are widely distributed among pathogenic *E. coli* strains, irrespective of the pathotype; (ii) some duplicated genes are only present in specific pathotypes; and (iii) some duplicated genes are strain specific. The present study also shows a relationship between duplications of both genes encoding regulators and genes encoding their targets. Our study also shows novel relationships between *E. coli* pathotypes. Finally, the distribution of duplicated genes in a high percentage of pathogenic *E. coli* isolates suggests that these genes must play a role in virulence. Hence, some of their gene products can serve as new targets for combating *E. coli* infections.

## Additional files

Additional file 1: **Table S1.** List of *E. coli* strains whose genomes have been used. **Table S2.** Distribution of genes from the *flu yeeR irmA aec70 aec71.* **Table S3.** Locus tag and gene function of each of the duplicated genes in regions 1, 2 and 3 of strain 042. **Table S4.** Locus tag and gene function of each of the duplicated genes in regions 1, 2, 3, 4, 5 and 6 of strain CFT073. **Table S5.** Locus tag and gene function of each of the duplicated genes in regions 1, 2, 3, 4, 5 and 6 of strain O145:H8. The locus tags of the different copies are shown. **Figure S1.** Five-set Venn diagram of the exclusive core-genome of the *hha*2/3^+^ set (*E. coli* strains 042, NA114, O104:H4 LB226692, ETEC H10407 and UMN026). **Figure S2.** Genes duplicated in the *E. coli* strain 042, identified by using BLASTn instead of BLASTp. **Figure S3.** Distribution of the strain CFT073 duplicated genes in other *E. coli* strains belonging to a wide range of pathotypes. **Figure S4.** Distribution of strain O145:H28 duplicated genes in other *E. coli* strains belonging to a wide range of pathotypes. (DOC 2532 kb)

Additional file 2: DNA sequences of the genes comprising the three shared families identified in the exclusive core genome of the *hha2/hha3*^*+*^ set (strains 042, NA114, O104:H4 2011C-3493, ETEC H10407 and UMN026). (DOCX 73 kb)

## Abbreviations

BLAST: Basic Local Alignment Search Tool
*E. coli*: *Escherichia coli*
EAEC: Enteroaggregative *Escherichia coli*
EHEC: Enterohemorrhagic *Escherichia coli*
EIEC: Enteroinvasive *Escherichia coli*
EPEC: Enteropathogenic *Escherichia coli*
ETEC: Enterotoxigenic *Escherichia coli*
UPEC: Uropathogenic *Escherichia coli*

## References

[1] Kaper JB, Nataro JP, Mobley HL (2004). Pathogenic *Escherichia coli*. Nat Rev Microbiol.

[2] Nataro JP, Kaper JB, Robins-Browne R, Prado V, Vial P, Levine MM (1987). Patterns of adherence of diarrheagenic *Escherichia coli* to HEp-2 cells. Pediatr Infect Dis J.

[3] Okeke IN, Wallace-Gadsden F, Simons HR, Matthews N, Labar AS, Hwang J (2010). Multi-locus sequence typing of enteroaggregative *Escherichia coli* isolates from Nigerian children uncovers multiple lineages. PLoS One.

[4] Frank C, Werber D, Cramer JP, Askar M, Faber M, an der Heiden M (2011). Epidemic profile of Shiga-toxin-producing *Escherichia coli* O104:H4 outbreak in Germany. N Engl J Med.

[5] Bielaszewska M, Mellmann A, Zhang W, Köck R, Fruth A, Bauwens A (2011). Characterisation of the *Escherichia coli* strain associated with an outbreak of haemolytic uraemic syndrome in Germany, 2011: a microbiological study. Lancet Infect Dis.

[6] Mayer CL, Leibowitz CS, Kurosawa S, Stearns-Kurosawa DJ (2012). Shiga toxins and the pathophysiology of hemolytic uremic syndrome in humans and animals. Toxins (Basel).

[7] Nataro JP, Kaper JB (1998). Diarrheagenic *Escherichia coli*. Clin Microbiol Rev.

[8] Nataro JP, Deng Y, Cookson S, Cravioto A, Savarino SJ, Guers LD (1995). Heterogeneity of enteroaggregative *Escherichia coli* virulence demonstrated in volunteers. J. Infect. Dis.

[9] Chaudhuri RR, Sebaihia M, Hobman JL, Webber MA, Leyton DL, Goldberg MD (2010). Complete genome sequence and comparative metabolic profiling of the prototypical enteroaggregative *Escherichia coli* strain 042. PLoS One.

[10] Nataro JP, Scaletsky IC, Kaper JB, Levine MM, Trabulsi LR (1985). Plasmid-mediated factors conferring diffuse and localized adherence of enteropathogenic *Escherichia coli*. Infect Immun.

[11] Nataro JP, Yikang D, Yingkang D, Walker K (1994). AggR, a transcriptional activator of aggregative adherence fimbria I expression in enteroaggregative *Escherichia coli*. J Bacteriol.

[12] Czeczulin JR, Balepur S, Hicks S, Phillips A, Hall R, Kothary MH (1997). Aggregative adherence fimbria II, a second fimbrial antigen mediating aggregative adherence in enteroaggregative *Escherichia coli*. Infect Immun.

[13] Morin N, Tirling C, Ivison SM, Kaur AP, Nataro JP, Steiner TS (2010). Autoactivation of the AggR regulator of enteroaggregative *Escherichia coli* in vitro and in vivo. FEMS Immunol Med Microbiol.

[14] Prieto A, Urcola I, Blanco J, Dahbi G, Muniesa M, Quirós P (2016). Tracking bacterial virulence: global modulators as indicators. Sci Rep.

[15] Madrid C, Balsalobre C, García J, Juárez A (2007). The novel Hha/YmoA family of nucleoid-associated proteins: use of structural mimicry to modulate the activity of the H-NS family of proteins. Mol Microbiol.

[16] Madrid C, García J, Pons M, Juárez A (2007). Molecular evolution of the H-NS protein: interaction with Hha-like proteins is restricted to enterobacteriaceae. J Bacteriol.

[17] Shintani M, Suzuki-Minakuchi C, Nojiri H (2015). Nucleoid-associated proteins encoded on plasmids: occurrence and mode of function. Plasmid..

[18] Zhang J (2003). Evolution by gene duplication: an update. Trends Ecol Evol.

[19] He X, Zhang J (2005). Gene complexity and gene duplicability. Curr Biol.

[20] Conant GC, Wolfe KH (2008). Turning a hobby into a job: how duplicated genes find new functions. Nat. Rev. Genet..

[21] Serres MH, Kerr ARW, McCormack TJ, Riley M (2009). Evolution by leaps: gene duplication in bacteria. Biol Direct.

[22] Innan H, Kondrashov F (2010). The evolution of gene duplications: classifying and distinguishing between models. Nat Rev Genet.

[23] Gao Y, Zhao H, Jin Y, Xu X, Han G-Z (2017). Extent and evolution of gene duplication in DNA viruses. Virus Res.

[24] Miele V, Penel S, Duret L (2011). Ultra-fast sequence clustering from similarity networks with SiLiX. BMC Bioinformatics.

[25] Altschul SF, Gish W, Miller W, Myers EW, Lipman DJ (1990). Basic local alignment search tool. J Mol Biol.

[26] R Development Core Team (2008). R-project.org. Vienna, Austria; http://www.R-project.org

[27] Prieto A, Bernabeu M, Aznar S, Ruiz-Cruz S, Bravo A, Queiroz MH, et al. Evolution of bacterial global modulators: role of a novel H-NS paralogue in the Enteroaggregative *Escherichia coli* strain 042. MSystems. 2018;3(3):e00220–17. 10.1128/mSystems.00220-17 PMC5861252 29577085

[28] Moriel DG, Heras B, Paxman JJ, Lo AW, Tan L, Sullivan MJ (2016). Molecular and structural characterization of a novel *Escherichia coli* interleukin receptor mimic protein. MBio..

[29] Walsh JB (1995). How often do duplicated genes evolve new functions?. Genetics..

[30] Arun PVPS, Miryala SK, Chattopadhyay S, Thiyyagura K, Bawa P, Bhattacharjee M (2016). Identification and functional analysis of essential, conserved, housekeeping and duplicated genes. FEBS Lett.

[31] Kondrashov FA (2012). Gene duplication as a mechanism of genomic adaptation to a changing environment. Proc Biol Sci.

[32] Elliott KT, Cuff LE, Neidle EL (2013). Copy number change: evolving views on gene amplification. Future Microbiol.

[33] Hooper SD, Berg OG (2003). Duplication is more common among laterally transferred genes than among indigenous genes. Genome Biol.

[34] Restieri C, Garriss G, Locas M-C, Dozois CM (2007). Autotransporter-encoding sequences are phylogenetically distributed among *Escherichia coli* clinical isolates and reference strains. Appl Environ Microbiol.

[35] Roche A, McFadden J, Owen P (2001). Antigen 43, the major phase-variable protein of the *Escherichia coli* outer membrane, can exist as a family of proteins encoded by multiple alleles. Microbiology (Reading, Engl).

[36] van der Woude MW, Henderson IR (2008). Regulation and function of Ag43 (*flu*). Annu Rev Microbiol.

[37] Regua-Mangia AH, Irino K, da Silva Pacheco R, Pimentel Bezerra RM, Santos Périssé AR, Teixeira LM (2010). Molecular characterization of uropathogenic and diarrheagenic *Escherichia coli* pathotypes. J Basic Microbiol.

[38] Lara FBM, Nery DR, de Oliveira PM, Araujo ML, Carvalho FRQ, Messias-Silva LCF (2017). Virulence markers and phylogenetic analysis of *Escherichia coli* strains with hybrid EAEC/UPEC genotypes recovered from sporadic cases of Extraintestinal infections. Front Microbiol.

[39] Zhang W, Sack DA (2015). Current Progress in developing subunit vaccines against Enterotoxigenic *Escherichia coli*-associated diarrhea. Clin Vaccine Immunol.

[40] Sullivan MJ, Petty NK, Beatson SA (2011). Easyfig: a genome comparison visualizer. Bioinformatics..

