# Supplementary material for: Gene duplications in the E. coli genome: common themes among pathotypes
Source: BMC Genomics. 2019 Apr 24;20:313. doi: 10.1186/s12864-019-5683-4 (PMC6480617; doi:10.1186/s12864-019-5683-4)
Supplement: Supplementary file 1 — Table S1. List of E. coli strains whose genomes have been used. Table S2. Distribution of genes from the flu yeeR irmA aec70 aec71. Table S3. Locus tag and gene function of each of the duplicated genes in regions 1, 2 and 3 of strain 042. Table S4. Locus tag and gene function of each of the duplicated genes in regions 1, 2, 3, 4, 5 and 6 of strain CFT073. Table S5. Locus tag and gene function of each of the duplicated genes in regions 1, 2, 3, 4, 5 and 6 of strain O145:H8. The locus tags of the different copies are shown. Figure S1. Five-set Venn diagram of the exclusive core-genome of the hha2/3+ set (E. coli strains 042, NA114, O104:H4 LB226692, ETEC H10407 and UMN026). Figure S2. Genes duplicated in the E. coli strain 042, identified by using BLASTn instead of BLASTp. Figure S3. Distribution of the strain CFT073 duplicated genes in other E. coli strains belonging to a wide range of pathotypes. Figure S4. Distribution of strain O145:H28 duplicated genes in other E. coli strains belonging to a wide range of pathotypes. (DOC 2532 kb) [file 12864_2019_5683_MOESM1_ESM.doc]

*Additional File 1*

**Gene duplications in the *E. coli* genome: common themes among pathotypes**

Bernabeu, M.^1^, Sanchez-Herrero, J.F.^1,2^, Huedo, P.^3^, Prieto, A.^1^, Hüttener, M.^1^, Rozas, J.^1,2^ and Juárez, A.^1, 4*^

^1^Department of Genetics, Microbiology and Statistics, University of Barcelona, Barcelona, Spain.

^2^Biodiversity Research Institute ([IRBio](http://www.ub.edu/irbio/index.php)), University of Barcelona, Barcelona, Spain.

# ^3^Institute of Biotechnology and Biomedicine (IBB), Universitat Autònoma de Barcelona, Cerdanyola del Vallès, Spain.

^4^Institute for Bioengineering of Catalonia, The Barcelona Institute of Science and Technology, Barcelona, Spain.

*Corresponding author: Prof. Antonio Juárez ([ajuarez@ub.edu)](mailto:ajuarez@ub.edu).

**Table S1.**

| **Pathotype** | **Strain** | **Presence of *hha2/3*** | **Accession number** | **Assembly Accesion** |
| --- | --- | --- | --- | --- |
| Commensal | *E. coli* K-12 MG1655 | *hha2/3^-^* | AJGD00000000.1 | GCF_000482265.1 |
| EAEC | *E.coli* 042 | *hha2/3^+^* | NC_017626.1 | GCF_000027125.1 |
|  | *E.coli* O104:H4 2011C-3493 | *hha2/3^+^* | NC_018658.1 | GCF_000299455.1 |
|  | *E. coli* O104:H4 LB226692 | *hha2/3*^+^ | AFOB00000000.2 | GCA_000215685.3 |
|  | *E.coli* 55989 | *hha2/3^+^* | NC_011748.1 | GCF_000026245.1 |
| EHEC | *E. coli* O26:H11 11368 | *hha2/3^+^* | NC_013361.1 | GCF_000091005.1 |
|  | *E. coli* O157:H7 Sakai | *hha2/3^-^* | NC_002695.1 | GCF_000008865.1 |
|  | *E. coli* O111:H- 11128 | *hha2/3^-^* | AP010960.1 | GCF_000010765.1 |
|  | *E. coli* O145:H28 RM13516 | *hha2^+^* | CP006262.1 | GCF_000520055.1 |
| ETEC | *E. coli* UMNF18 | *hha2/3^-^* | AGTD00000000.1 | GCF_000220005.1 |
|  | *E. coli* O139:H28 E24377A | *hha3^+^* | JXRF00000000.1 | GCF_000017745.1 |
|  | *E. coli* O103:H2 2011C-3750 | *hha2/3^-^* | JHLL00000000.1 | GCF_000616345.2 |
|  | *E. coli* H10407 | *hha3^+^* | FN649414.1 | GCF_000210475.1 |
| EPEC | *E. coli* O127:H6 E2348-69 | *hha2/3^-^* | NC_011601.1 | GCF_000026545.1 |
|  | *E. coli* O55:H7 CB9615 | *hha2^+^* | CP001846.1 | GCF_000025165.1 |
| EIEC | *E. coli* O96:H19 | *hha2^+^* | JHNY01000124.1 | GCF_001007915.1 |
|  | *E. coli* O143:H26 4608-58 | *hha2/3^+^* | JTCO01000000 | GCF_000805835.1 |
|  | *E. coli* O28ac:NM 02-3404 | *hha2/3^+^* | JHNY00000000 | GCF_000617165.2 |
|  | *E. coli* O124:H30 M4163 | *hha2/3^+^* | JTCN01000000 | GCF_000805815.1 |
|  | *E. coli* 53638 | *hha2/3^-^* | AAKB00000000.2 | GCF_000167915.2 |
| ST131 | *E. coli* JJ1886 | *hha2/3^+^* | CP006784.1 | GCF_000493755.1 |
|  | *E. coli* O25b:H4 EC958 | *hha2/3^-^* | HG941718.1 | GCF_000285655.3 |
|  | *E. coli* NA114 | *hha2/3^+^* | MIPU00000000.1 | GCF_000214765.2 |
| UPEC | *E. coli* 536 | *hha2/3^-^* | CP000247.1 | GCF_000013305.1 |
|  | *E. coli* UMN026 | *hha2^+^* | CU928163.2 | GCF_000026325.1 |
|  | *E. coli* CFT073 | *hha2/3^+^* | AE014075.1 | GCF_000007445.1 |
|  | *E. coli* UTI89 | *hha3^+^* | CP000243.1 | GCF_000013265.1 |
|  | *E. coli* IAI39 | *hha2/3^-^* | NC_011750.1 | GCF_000026345.1 |

**Table S1**. List of *E. coli* strains whose genomes have been used for the different genomic analysis performed in this work. Pathotype, accession number and GenBank assembly accession details are indicated.

**Table S2.**

| **Pathotype** | **strain/gene** | ***flu*** | ***yeeR*** | ***irmA*** | ***aec70*** | ***aec71*** | ***hha2/3*** |
| --- | --- | --- | --- | --- | --- | --- | --- |
| EAEC | *E. coli* 042 | X | X | X | X | X | *hha*2/3^+^ |
|  | *E. coli* O104_H4 2011C-3493 | X | X | X | X | X | *hha*2/3^+^ |
|  | *E. coli* O104:H4 LB226692 |  | X | X | X | X | *hha*2/3^+^ |
|  | *E. coli* 55989 | X | X | X | X | X | *hha*2/3^+^ |
| EHEC | *E. coli* O26_H11_11368 | X | X | X | X | X | *hha2/*3^+^ |
|  | *E. coli* O145_H28 RM13516 | X | X | X | X | X | *hha*2^+^ |
| ETEC | *E. coli* O139_H28_E24377A |  |  |  |  | X | *hha*3^+^ |
|  | *E. coli* ETEC_H10407 |  | X | X | X | X | *hha*3^+^ |
| EPEC | *E. coli* O55_H7_CB9615 |  |  |  |  | X | *hha*2^+^ |
| EIEC | *E. coli* O96-H19 |  |  |  |  |  | *hha*2^+^ |
|  | *E. coli* O143-H26_4608-58 | X | X |  |  | X | *hha2/*3^+^ |
|  | *E. coli* O28ac-NM_02-3404 | X | X | X |  | X | *hha2/*3^+^ |
|  | *E. coli* O124-H30_M4163 | X | X | X | X | X | *hha2/*3^+^ |
| ST131 | *E. coli* JJ1886 | X |  | X | X | X | *hha2/3*^+^ |
|  | *E. coli* NA114 |  | X | X | X | X | *hha*2/3^+^ |
| UPEC | *E. coli* UMN026 | X | X | X |  | X | *hha*2^+^ |
|  | *E. coli* CFT073 | X | X | X | X | X | *hha*2/3^+^ |
|  | *E. coli* UTI89 |  |  |  |  | X | *hha*3^+^ |
| Commensal | *E. coli* K-12_MG1655 | X |  |  |  |  | *hha*2/3^-^ |
| EHEC | *E. coli* O157_H7_Sakai |  |  |  |  | X | *hha*2/3^-^ |
|  | *E. coli* O111_H-11128 |  |  |  |  | X | *hha*2/3^-^ |
| ETEC | *E. coli* UMNF18 |  | X | X | X | X | *hha*2/3^-^ |
|  | *E. coli* O103-H2_2011C-3750 | X | X | X | X | X | *hha*2/3^-^ |
| EPEC | *E. coli* O127_H6_E2348-69 |  |  |  |  | X | *hha*2/3^-^ |
| EIEC | *E. coli* 53638 |  |  |  |  | X | *hha*2/3^-^ |
| ST131 | *E. coli* EC958 | X | X | X |  |  | *hha*2/3^-^ |
| UPEC | *E. coli* 536 | X |  |  |  | X | *hha*2/3^-^ |
|  | *E. coli* IAI39 |  |  |  |  | X | *hha*2/3^-^ |

**Table S2**. Distribution of genes from the *flu yeeR irmA aec70 aec71* gene cluster among the 28 *E. coli* strains studied in this work.

**Table S3.**

|  | **Group** | **Locus tag 1** | **Locus tag 2** | **Locus tag 3** | **Locus tag 4** | **Description** |
| --- | --- | --- | --- | --- | --- | --- |
| **Region 1: 21-35** | 21 | EC042_1328 | EC042_2193 |  |  | putative phage protein |
|  | 22 | EC042_1329 | EC042_2192 |  |  | putative phage protein |
|  | 23 | EC042_1330 | EC042_2191 |  |  | putative phage protein |
|  | 24 | EC042_1333 | EC042_2189 |  |  | phage protein |
|  | 25 | EC042_1336 | EC042_1705 | EC042_2186 |  | putative host cell_killing modulation protein |
|  | 26 | EC042_1342 | EC042_2183 |  |  | phage protein |
|  | 27 | EC042_1343 | EC042_2182 |  |  | putative phage endodeoxyribonuclease |
|  | 28 | EC042_1344 | EC042_2181 |  |  | phage antitermination protein |
|  | 29 | EC042_1349 | EC042_1703E | EC042_2175 |  | putative phage lysozyme |
|  | 30 | EC042_1353 | EC042_1702 |  |  | putative phage protein |
|  | 31 | EC042_1371 | EC042_1685 |  |  | phage minor tail protein |
|  | 32 | EC042_1372 | EC042_1509 | EC042_1684 | EC042_2138 | phage minor tail protein |
|  | 33 | EC042_1373 | EC042_1510 | EC042_1683 | EC042_2137 | phage tail assembly protein |
|  | 34 | EC042_1376 | EC042_1512 | EC042_2135 |  | phage host specificity protein |
|  | 35 | EC042_1377 | EC042_1513 | EC042_1680 | EC042_2134 | putative prophage_encoded outer membrane protein |
| **Region 2: 46-60** | 46 | EC042_2236A | EC042_4519 | EC042_4793 |  | conserved hypothetical protein |
|  | 47 | EC042_2237 | EC042_4518 | EC042_4794 |  | conserved hypothetical protein |
|  | 48 | EC042_2238 | EC042_4795 |  |  | hypothetical protein |
|  | 49 | EC042_2239 | EC042_4798 |  |  | conserved hypothetical protein |
|  | 50 | EC042_2241 | EC042_4512 | EC042_4802 |  | putative GTP_binding protein |
|  | 51 | EC042_2242 | EC042_4511 |  |  | antigen 43 precursor (fluffing protein) (autotransporter) |
|  | 52 | EC042_2243 | EC042_4510 |  |  | putative membrane protein |
|  | 53 | EC042_2244 | EC042_4509 |  |  | putative exported protein |
|  | 54 | EC042_2244A | EC042_4508A |  |  | conserved hypothetical protein |
|  | 55 | EC042_2245 | EC042_3221 | EC042_4507 | EC042_4805 | conserved hypothetical protein |
|  | 56 | EC042_2246 | EC042_3222 | EC042_4506 |  | putative antirestriction protein |
|  | 57 | EC042_2247 | EC042_3223 | EC042_4505 | EC042_4807 | putative DNA repair protein |
|  | 58 | EC042_2247A | EC042_3224 | EC042_4504 | EC042_4808 | conserved hypothetical protein |
|  | 59 | EC042_2248 | EC042_3225 | EC042_4503 |  | conserved hypothetical protein |
|  | 60 | EC042_2249 | EC042_3226 | EC042_4502 |  | conserved hypothetical protein |
| **Region 3: 64-71** | 64 | EC042_3180 | EC042_4556 |  |  | conserved hypothetical protein |
|  | 65 | EC042_3181 | EC042_4555 |  |  | putative transcriptional regulator |
|  | 66 | EC042_3182 | EC042_4554 |  |  | ParB_like nuclease |
|  | 67 | EC042_3183 | EC042_4553 |  |  | conserved hypothetical protein |
|  | 68 | EC042_3187 | EC042_4548 |  |  | putative helicase |
|  | 69 | EC042_3189 | EC042_4523 |  |  | phage protein |
|  | 70 | EC042_3190 | EC042_4522 |  |  | conserved hypothetical protein |
|  | 71 | EC042_3191 | EC042_4521 |  |  | putative DNA_binding protein |

**Table S3.** Locus tag and gene function of each of the duplicated genes in regions 1, 2 and 3 of strain 042. The locus tags of the different copies are shown. Gene functions correspond to locus tag 1.

**Table S4.**

**Table S5.** Locus tag and gene function of each of the duplicated genes in regions 1, 2, 3, 4, 5 and 6 of strain CFT073. The locus tags of the different copies are shown. Gene functions correspond to locus tag **Table S4.** Locus tag and gene function of each of the duplicated genes in regions 1, 2, 3, 4, 5 and 6 of strain CFT073. The locus tags of the different copies are shown. Gene functions correspond to locus tag 1.

**Table S5.**

**Table S5.** Locus tag and gene function of each of the duplicated genes in regions 1, 2, 3, 4, 5 and 6 of strain O145:H8. The locus tags of the different copies are shown. Gene functions correspond to locus tag 1.

**
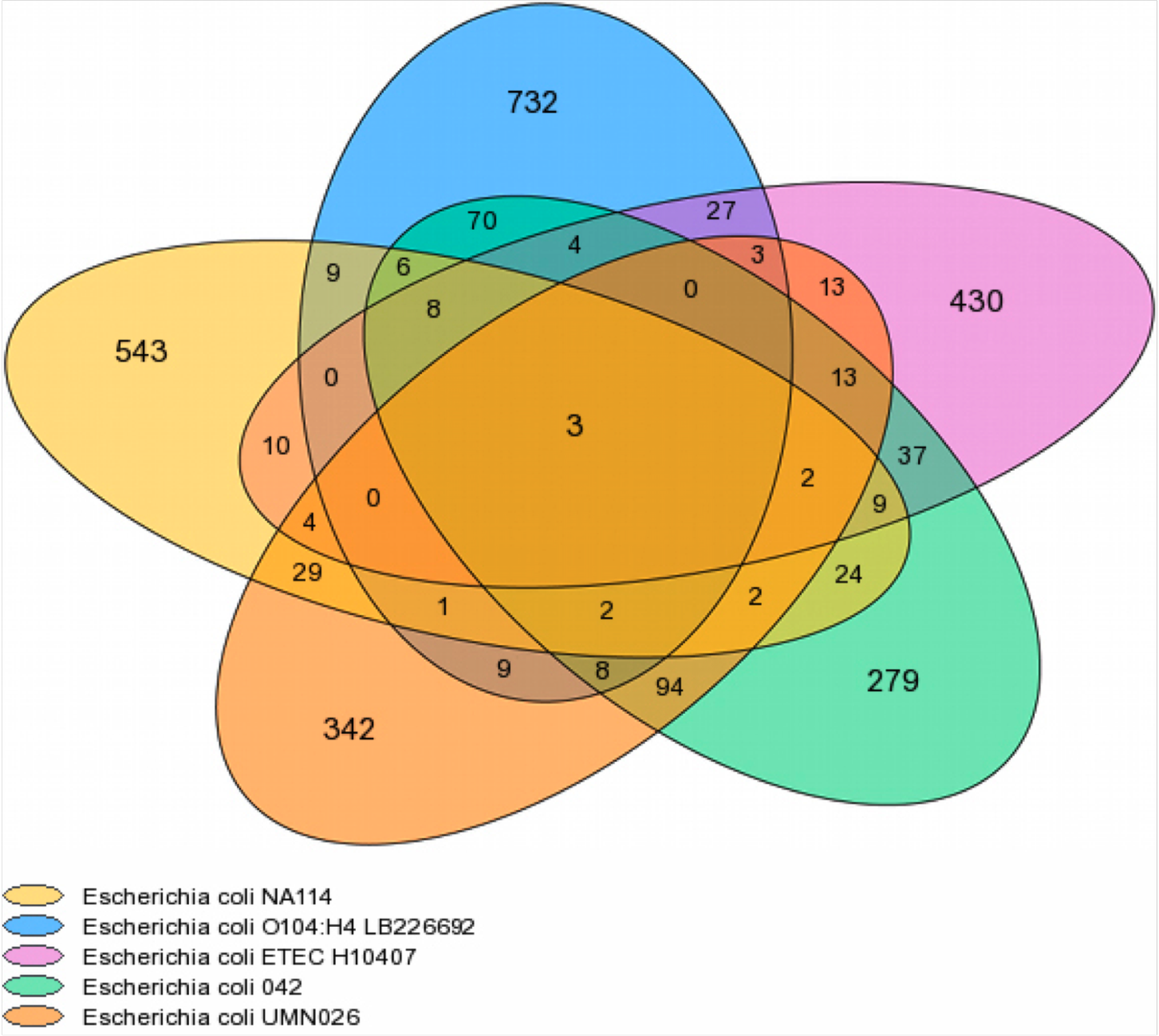
**

**Figure S1.** Five-set Venn diagram of the exclusive core-genome of the *hha*2/3^+^ set (*E. coli* strains 042, NA114, O104:H4 LB226692, ETEC H10407 and UMN026). Each ellipse displays the total number of coding sequences corresponding to a gene family of each *hha2/3^+^* strain which is not present in any genome of the five *hha2/3^-^* set (*E. coli* strains O111:H- 11128, 53638, IAI39, O127:H6 E2348/69 and O157:H7 Sakai). Intersections indicate shared genes between strains in a set. As shown, only three gene families are common to the five-strain set. No gene family of the *hha2/3^-^* set was exclusive and was present in the *hha2/3^+^* set (not shown in the figure).


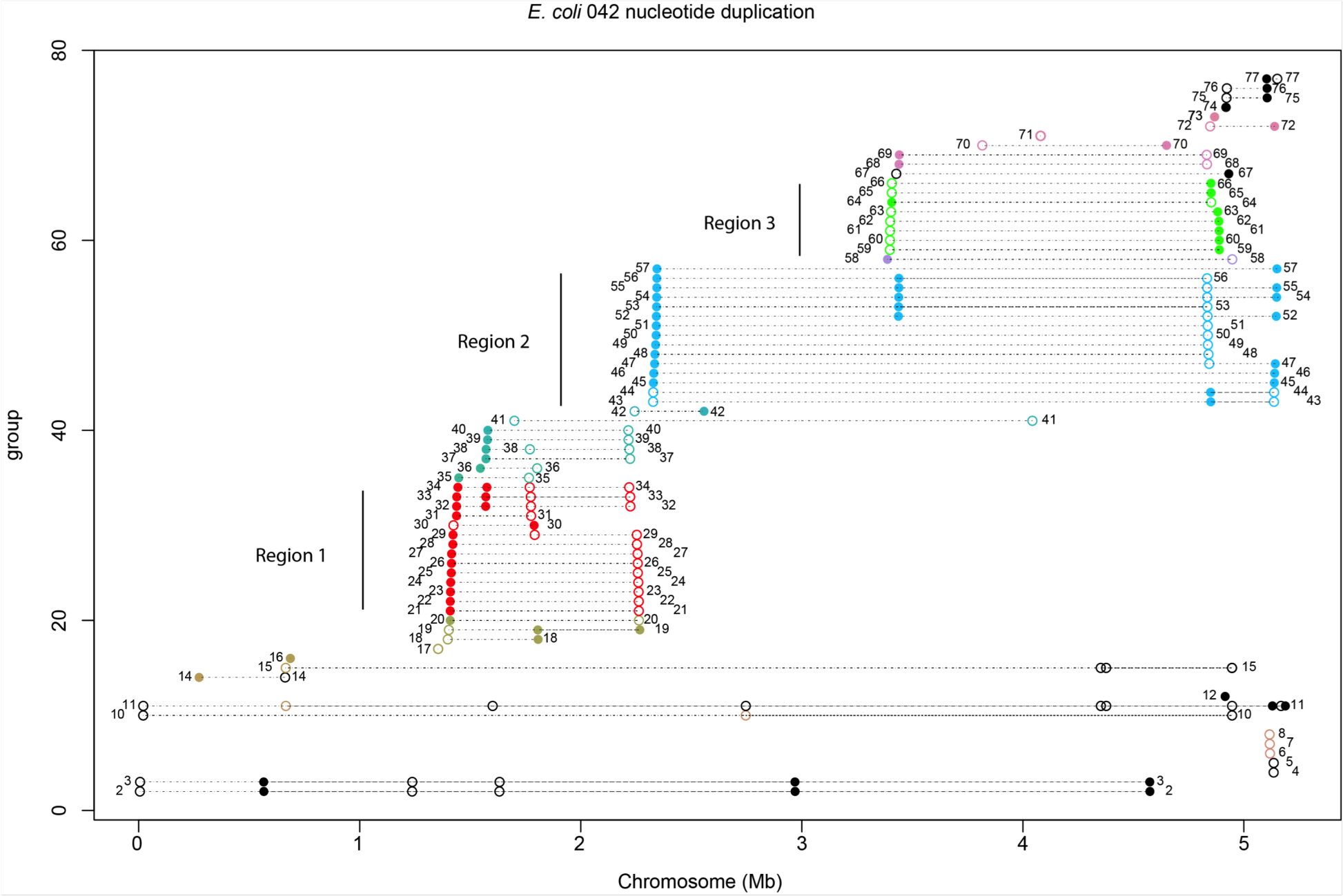


**Figure S2**. Genes duplicated in the *E. coli* strain 042, identified by using BLASTn instead of BLASTp. The X axis corresponds to the lineal map of the chromosome. Each group of spots connected by a horizontal dashed line corresponds to a single gene duplicated or amplified in different positions of the chromosome. The different spots indicate the map position of the different copies of the gene. Point shapes represent the strand on which a protein is codified: filled circle for (+) strand and circle for (-) strand. Numbers correspond to the different duplicated genes, which have been numbered by their order starting from the origin of the chromosomal map. Black closed and open circles correspond to transposases. Colors and vertical bars define the three main regions that contain duplicated genes.


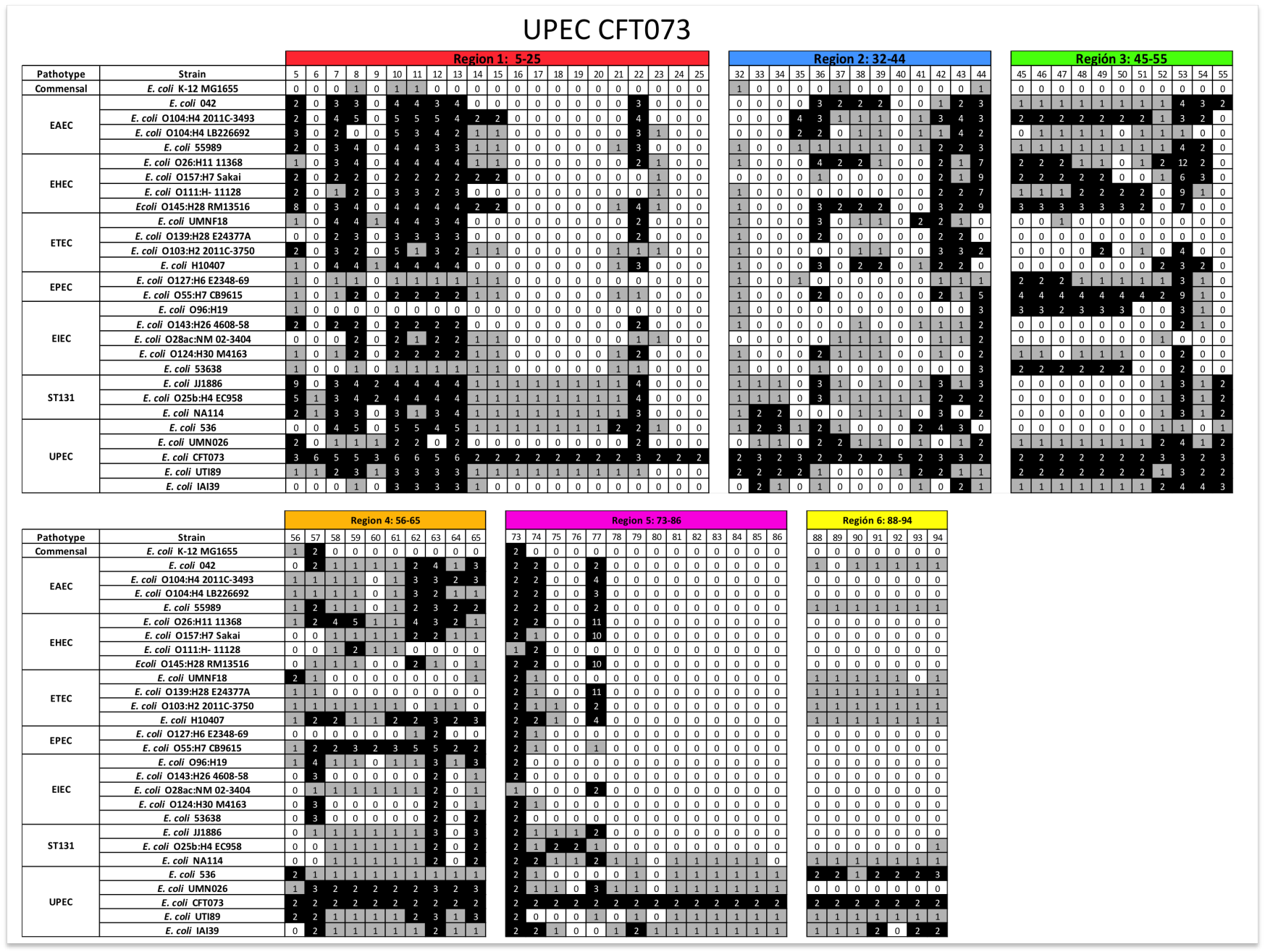


**Figure S3.** Distribution of the strain CFT073 duplicated genes in other *E. coli* strains belonging to a wide range of pathotypes. White color, gene absent. Grey color, gene present in a single copy. Black color, gene amplified. The numbers show the extent of gene duplication. Colours correspond to the identified regions.

**
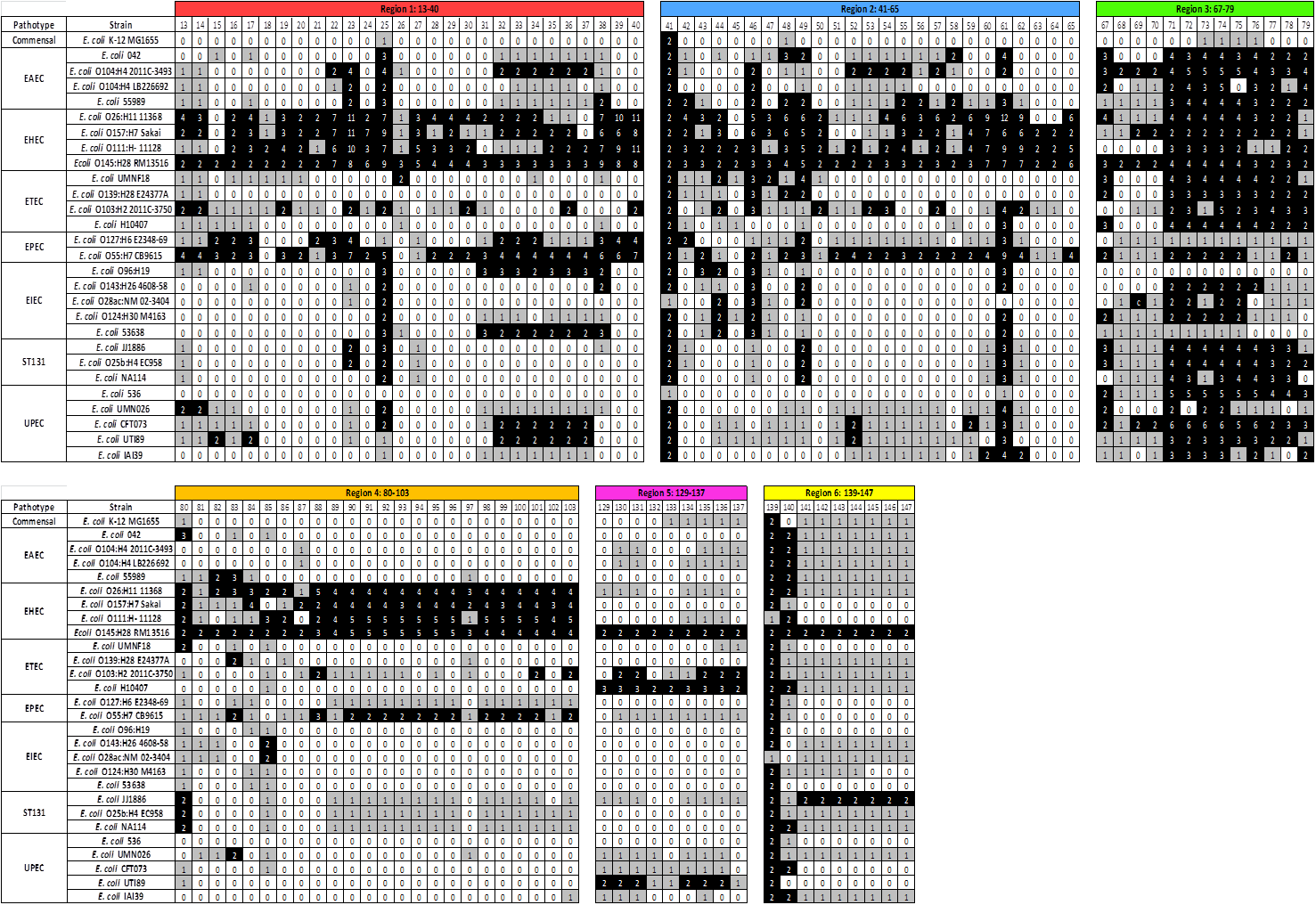
**

**Figure S4.** Distribution of strain O145:H28 duplicated genes in other *E. coli* strains belonging to a wide range of pathotypes. White color, gene absent. Grey colur, gene present in a single copy. Black color, gene amplified. The numbers show the extent of gene amplification. Colors correspond to the identified regions.
