# Supplementary material for: Gene duplications in the E. coli genome: common themes among pathotypes
Source: BMC Genomics. 2019 Apr 24;20:313. doi: 10.1186/s12864-019-5683-4 (PMC6480617; doi:10.1186/s12864-019-5683-4)
Supplement: Supplementary file 2 — DNA sequences of the genes comprising the three shared families identified in the exclusive core genome of the hha2/hha3+ set (strains 042, NA114, O104:H4 2011C-3493, ETEC H10407 and UMN026). (DOCX 73 kb) [file 12864_2019_5683_MOESM2_ESM.docx]

*Additional File 2*

**Gene duplications in the *E. coli* genome: common themes among pathotypes**

Bernabeu, M.^1^, Sanchez-Herrero, J.F.^1,2^, Huedo, P.^3^, Prieto, A.^1^, Hüttener, M.^1^, Rozas, J.^1,2^ and Juárez, A.^1, 4*^

^1^Department of Genetics, Microbiology and Statistics, University of Barcelona, Barcelona, Spain.

^2^Biodiversity Research Institute ([IRBio](http://www.ub.edu/irbio/index.php)), University of Barcelona, Barcelona, Spain.

# ^3^Institute of Biotechnology and Biomedicine (IBB), Universitat Autònoma de Barcelona, Cerdanyola del Vallès, Spain.

^4^Institute for Bioengineering of Catalonia, The Barcelona Institute of Science and Technology, Barcelona, Spain.

*Corresponding author: Prof. Antonio Juárez ([ajuarez@ub.edu)](mailto:ajuarez@ub.edu).

DNA sequences of the genes conforming the three-shared families identified in the exclusive core-genome of the *hha2/hha3^+^* set (strains 042, NA114, O104:H4 2011C-3493, ETEC H10407 and UMN026).

**>6703|3055749|ECUMN_3401|conserved hypothetical protein; putative membrane protein, yeeR [Escherichia coli UMN026]**

GTGACTATGTTACAGATAGTCGGCGCGCTGATTCTGCTGATCGCAGGATTTGCCATTCTT

CGCCTTTTGTTCAGAGCATTAACCAGCACAGCGTCTGCGCTGGCAGGGTTCATATTGCTG

TGTCTGTTCGGTCCGGCTTTACTGGCTGGCTATATCACTGAACGCATAACCCGGTTATTC

CATATTCGCTGGCTGGCAGGCGTATTTCTGACGATTGCCGGAATGATCATCAGCTTCATG

TGGGGACTTGATGGTAAACATATCGCACTGGAGGCTCATACTTTTGACTCTGTAAAATTT

ATTCTGACCACCGCTCTCGCCGCTGGTCTGCTGGCTCTTCCCGTGCAGATAAGAACCATT

CAGCAGAGCGGGCTCACACCTGAAGATATCAGCAAGGAAATTAACGGGTATTACTGCTGT

TTTTATACTGCTTTTTTCCTTATGGCGTGTTCTGCATACGCACCATTGATCGCATTGCAG

TTCGATATTTCACCCTCACTGATGTGGTGGGGCGGGTTGTTGTACTGGCTGGCTGCATTA

GTGACGCTGCTATGGGCGGCCAGCCAGATCCAGGCGCTGAAAAGACTGACCAGTGCCATC

CGCCAGACACTGGAAGAACAACCGGTGCTCAACAGTAAATCGTGGCTGAGCAGTTTGCAA

AACGATTACAGCCTTCCTGAAACGCTGACGGAGCGCATCTGGCTGACACTCATTTCACAA

CGGATTTCCCGGGGAGAACTGAGGGAATTTGAACTGGCAGACGGAAACTGGCTACTGGAC

AATGCCTGGTATGAAAGAAACATGGCGGGTTTCAACGAAAAGCTGAGAGAGAGCCTGTCA

TTTACCCCTGATGAACTGAAAACCCTCTTCCGGAACCGCCTGAATTTATCACCGGAAGCG

AATGACGATTTTCTCGATCGTTGCCTGGACGGCGGTGACTGGTACCCCTTTTCAGAAGGC

CGCCGTTTTGTATCATTCCACCACGTGGATGAGCTTCGTATCTGTGCCTCCTGCGGGCTG

ACAGAAGTACATCATGCCCCGGAAAATCATAAGCCGGATCCGGAATGGTACTGCTCCTCT

CTTTGTCGCGAAACAGAAACACTGTGTCAGGACATTTATGAACGTTCTTACACCGGTTTT

ATTTCCGATGCAACGGCGAATGGTCTGATTCTCATGAAACTGCCGGAAACCTGGAGTACA

AATGAGAAAATGTTTGCTTCCGGAGGGCAGGGACATGGGTTTGCCGCTGAACGGGGAAAC

CATATTGTCGACAGAGTCCGTCTGAAAAACGCACGGATCCTCGGTGATAATAATGCCAGA

AATGGAGCAGACAGACTGGTCAGCGGAACAGAAATCCAGACGAAATATTGTTCAACTGCA

GCCCGTAGCGTCGGTGCGGCATTCGACGGACAAAACGGACAGTATCGTTACATGGGAAAT

CATGGTCCCATGCAACTGGAAGTCCCCCGTGATCAGTATGCCGGCGCTGTGGAAACCATG

AAGAATAAGATCCGCGAAGGTAAAGTACCCGGTGTAACCGATCCCGCAGAAGCGTCCCGG

CTGATTCGTCGGGGACATCTGACTTATACCCAGGCCCGTAATATCACCCGGTTCGGGACC

ATCGAATCGGTCACTTATGATATTGCCGAGGGGTCGGTTATCAGTCTGGCGGCCGGAGGG

ATCAGTTTTGCCCTGACGGCATCGGTCTTCTGGCTCAGCACCGGCGATCGCGATGCTGCC

CTGCAGACAGCTGCTGTCCAGGCAGGAAAAACCTTCACCCGCACACTGGCTGTCTACGTC

ACAACCCAGCAACTTCACCGGCTCACTGTTGTTCAGGGTATGCTGAAGCATATTGATTTT

TCGACGGCCAGCCCGACTGTCCGGCAGGCGCTTCAGAAGGGGACCGGTGCAGGAAATATC

AGTGCCCTGAACAAAGTGATGAAGGGTACGCTGGTGACATCTCTGGCACTGGTAGCTGTC

ACAACCGGCCCTGACATGATCAAAATGTTGCGGGGACGGATCTCCGGTGCGCAGTTCATC

AGGAATCTTGCCGTGGCATCTTCCGGTGTGGCAGGTGGTGCTGTCGGGTCAGTGGCGGGC

GGGATACTGTTCAGTCCACTGGGACCATTTGGTGCACTGACAGGGCGTGTGGTTGGCGGT

GTTCTGGGGGGAATGATTGCCTCCGCTGTATCAGGAAAAATTGCCGGAGCGCTGGTTGAA

GAAGATCGCGTCAAAATTCTGGCAATGATTCAGGAGCAGGTGACATGGCTTGCCGGCAGT

TTCCTGCTGACCGGACATGAGATTGAAAATCTGAACGCGAATCTGGCCCGTGTTATCGAT

CAGAATGCTCTGGAGATCATTTTCGCCGCCGGTATACAACAACGGGCCGCGACCAATATG

TTAATCAAACCACTGGTGGTCAGTATCATCAGGCAACGCCCCGTCATGGAATATGATGCA

TCCCATCTCGGCAATATGGTTAACCGACTGGAAGAAGCATTACCCCCGGAGTTACCGGCA

TAA

**>6703|938002|EC42_4699|conserved hypothetical protein; putative membrane protein, yeeR [Escherichia coli 042]**

GTGACTATGTTACAGATAGTCGGCGCGCTGATTCTGCTGATCGCAGGATTTGCCATTCTT

CGCCTTTTGTTCAGAGCATTAACCAGCACAGCGTCTGCGCTGGCAGGGTTCATATTGCTG

TGTCTGTTCGGTCCGGCTTTACTGGCTGGCTATATCACTGAACGCATAACCCGGTTATTC

CATATTCGCTGGCTGGCAGGCGTATTTCTGACGATTGCCGGAATGATCATCAGCTTCATG

TGGGGACTTGATGGTAAACATATCGCACTGGAGGCTCATACTTTTGACTCTGTAAAATTT

ATTCTGACCACCGCTCTCGCCGCTGGTCTGCTGGCTCTTCCCGTGCAGATAAGAACCATT

CAGCAGAACGGGCTCACACCTGAAGATATCAGCAAGGAAATTAACGGGTATTACTGCTGT

TTTTATACTGCCTTTTTCCTTATGGCGTGTTCTGCATACGCACCATTGATCGCATTGCAG

TTCGATATTTCACCCTCACTGATGTGGTGGGGCGGGTTGTTGTACTGGCTGGCTGCATTA

GTGACGCTGCTATGGGCGGCCAGCCAGATCCAGGCGCTGAAAAGACTGACCAGTGCCATC

AGCCAGACACTGGAAGAACAACCGGTGCTCAACAGTAAATCATGGCTGAGCAGTTTGCAA

AACGATTACAGCCTTCCTGAAACGCTGACGGAGCGCATCTGGCTGACACTCATTTCACAA

CGGATTTCCCGGGGAGAACTGAGGGAATTTGAACTGGCAGACGGAAACTGGCTACTGGAC

AATGCCTGGTATGAAAGAAACATGGCGGGTTTCAACGAAAAGCTGAGAGAGAGCCTGTCA

TTTACCCCTGATGAACTGAAAACCCTCTTCCGGAACCGCCTGAATTTATCACCGGAAGCG

AATGACGATTTTCTCGATCGTTGCCTGGACGGCGGTGACTGGTATCCCTTTTCAGAAGGT

CGCCGTTTTGTATCATTCCATCACGTGGATGAGCTTCGTATCTGTGCCTCCTGCGGGCTG

ACAGAAGTACATCATGCCCCGGAAAATCATAAGCCGGATCCGGAATGGTACTGCTCCTCT

CTTTGTCGCGAAACAGAAACACTGTGTCAGGAAATTTATGAACGCCCCTACAACAGTTTT

ATTTCCGATGCAACGGCGAATGGTCTGATTCTCATGAAACTGCCGGAAACCTGGAGTACA

AATGAGAAAATGTTTGCTTCCGGAGGGCAGGGACATGGGTTTGCCGCTGAACGGGGAAAC

CATATTGTCGACAGAGTCCGTCTGAAAAACGCACGGATCCTCGGTGATAATAATGCCAGA

AATGGAGCAGACAGACTGGTCAGCGGAACAGAGATCCAGACGAAATATTGTTCAACTGCA

GCCCGTAGCGTCGGTGCGGCATTCGACGGACAAAACGGACAGTATCGTTACATGGGAAAT

CATGGCCCCATGCAACTGGAAGTCCCCCGTGATCAGTATGCCGGCGCTGTTGAAACCATG

AGGAATAAGATCCGCGAAGGTAAAGTACCTGGTGTAACCGATCCCGCAGAAGCGTCCCGG

CTGATTCGTCGGGGACATCTGACTTATACCCAGGCCCGTAATATCACCCGGTTCGGGACC

ATCGAATCGGTCACTTATGATATTGCCGAGGGTTCGGTTGTCAGTCTGGCGGCCGGAGGG

ATCAGTTTTGCCCTGACGGCATCGGTCTTCTGGCTCAGCACCGGCGATCGCGATGCTGCC

CTGCAGACAGCTGCTGTCCAGGCAGGAAAAACCTTCACCCGCACACTGGCTGTCTACGTC

ACAACCCAGCAACTTCACCGGCTCAGTGTTGTTCAGGGTATGCTGAAGCACATTGATTTT

TCGACGGCCAGTCCGACTGTCCGACTGGCTCTTCAGAAGGGGACCGGTACAGGAAATATC

AGTGCCCTGAACAAAGTGATGAAGGGTACGCTGGTGACATCTCTGGCACTGGTAGCTGTC

ACAACCGGCCCTGACATGATCAAAATGTTGCGGGGACGGATCTCCGGTACGCAGTTCATC

AGGAATCTTGCCGTGGCATCTTCCGGTGTGGCAGGTGGAGCTGTCGGGTCAGTGGCGGGC

GGGATACTGTTCAGTCCACTGGGACCATTTGGTGCACTGACAGGGCGTGTGGTTGGCGGT

GTTCTGGGGGGAATGATTGCCTCCGCTGTATCAGGAAAAATTGCCGGAGCGCTGGTTGAA

GAAGATCGCGTCAAAATTCTGGCAATGATTCAGGAACAGGTGACATGGCTTGCCGGCAGT

TTCCTGCTGACCGGACATGAAACAGAAAATCTGAACGCGAATCTGGCCCGTGTTATCGAT

CAGAATGCTCTGGAGATCATTTTCGCCGCCGGTATACAACAACGGGCCGCGACCAATATG

TTAATCAAACCACTGGTGGTCAGTATCATCAGGCAACGCCCCGTCATGGAATATGATGCA

TCCCATCTCGGCAATATGGTTAACCGGCTGGAAGAAGCATTACCCCCGGAGTTACCGGCA

TAA

**>6703|935626|EC42_2323|conserved hypothetical protein; putative membrane protein, yeeR [Escherichia coli 042]**

GTGACTATGTTACAGATAGTCGGCGCGCTGATCCTGCTGATCGCAGGATTTGCCATTCTT

CGCCTTTTGTTCAGAGCATTAATCAGCACGGCTTCTGCGCTGGCAGGGCTCATATTGCTG

TGTCTGTTCGGCCCGGCCTTACTGGCTGGCTATATCACCGAACGCATAACCCGGTTGTTC

CATATTCGCTGGCTGGCAGGCGTATTTCTGACGATTGCCGGAATGATCATCAGCTTCATG

TGGGGACTTGATGGTAAACATATCGCGCTGGAGGCTCACACCTTTGACTCTGTGAAATTT

ATTCTGACCACCGCTCTCGCCGGTGGTCTGCTGGCTGTTCCCCTGCAGATCAAAAACATT

CAGCAGAACGGGATCACACCAGAAGATATCAGCAAGGAAATTAACGGGTATTACTGCTGT

TTTTATACTGCCTTTTTCCTTATGGCGTGTTCTGCATGCGCACCATTGATCGCGTTACAG

TACGATATTTCACCGTCACTGATGTGGTGGGGCGGGTTGTTGTACTGGCTGGCTGCATTA

GTGACGCTGCTATGGGCGGCCAGCCAAATCCAGGCGCTGAAAAAACTGACCTGTGCCATC

AGCCAGACACTGGAAGAACAACCGGTGCTCAACAGTAAATCGTGGCTGACCAGTTTGCAA

AACGATTACAGCCTTCCTGACTCACTGACGGAGCGCATCTGGCTGACGCTCATTTCTCAA

CGGATTTCCCGGGGAGAGCTGAGGGAATTTGAACTGGCAGACGGAAACTGGTTACTGAAC

AATGCCTGGTATGAAAGAAACATGGCAGGGTTTAACGAACAGTTGAAAGAGAACCTGTCA

TTCACACCTGATGAACTGAAAACGCTCTTCCGGAACCGCCTGAATTTATCACCGGAAGCG

AATGACGATTTTCTCGATCGTTGCCTGGACGGCGGTGACTGGTATCCCTTTTCAGAAGGT

CGCCGTTTTGTATCATTCCATCACGTGGATGAGCTTCGTATCTGTGCCTCCTGCGGGCTG

ACAGAAGTACATCATGCCCCGGAAAATCATAAGCCGGATCCGGAATGGTACTGCTCCTCT

CTTTGCCGCGAAACAGAAACACTGTGTCAGGAAATTTATGAACGCCCTTACAACAGCTTT

ATTTCCGATGCAACGGCGAATGGTCTGATTCTCATGAAACTGCCGGAAACCTGGAGTACA

AATGAGAAAATGTTTGCTTCCGGAGGGCAGGGACATGGGTTTGCCGCTGAACGGGGAAAC

CATATTGTCGACAGAGTCCGTCTGAAAAACGCACGGATCCTCGGTGATAATAATGCCAGA

AATGGAGCAGACAGACTGGTCAGCGGAACAGAAATCCAGACGAAATATTGTTCAACTGCA

GCCCGTAGCGTCGGTGCGGCATTCGACGGACAAAACGGACAGTATCGTTACATGGGAAAT

AATGGCCCCATGCAACTGGAAGTCCCCCGTGATCAGTATGCCGGCGCTGTTGAAACCATG

AGGAATAAGATCCGCGAAGGTAAAGTACCCGGTGTAACCGATCCCGCAGAAGCGTCCCGG

CTGATTCGTCGGGGACATCTGACTTATACTCAGGCCCGTAATATCACCCGGTTCGGGACC

ATCGAATCGGTCACTTATGATATTGCCGAGGGTTCGGTTGTCAGTCTGGCGGCCGGAGGG

ATCAGTTTTGCCCTGACGGCATCGGTCTTCTGGCTCAGCACCGGCGATCGCGATGCTGCC

CTGCAGACAGCTGCTGTCCAGGCAGGAAAAACCTTCACCCGCACACTGGCTGTCTACGTC

ACAACCCAGCAACTCCACCGGCTCAGTGTTGTTCAGGGTATGCTGAAGCACATTGATTTT

TCGACTGCCAGCCCGACTGTCCGACTGGCTCTTCAGAAGGGGACCGGTGCAGGAAATATC

AGTGCCCTGAACAAAGTAATGAAGGGTACGCTGGTGACATCTCTGGCACTGGTAGCTGTC

ACAACCGGCCCTGACATGATCAAAATGTTGCGGGGACGGATCTCCGGTGCGCAGTTCATC

AGGAATCTTGCCGTGGCATCTTCCGGTGTGGCAGGTGGAGCTGTCGGGTCAGTGGCGGGC

GGGATACTGTTCAGTCCACTGGGACCATTTGGTGCACTGACAGGGCGTGTGGTTGGCGGT

GTTCTGGGGGGAATGATTGCCTCCGCTGTATCAGGAAAAATTGCCGGAGCGCTGGTTGAA

GAAGATCGCGTCAAAATTCTGGCAATGATTCAGGAGCAGGTGACATGGCTTGCCGGCAGT

TTCCTGCTGACCGGACATGAGATTGAAAATCTGAACGAGAATCTGGCCCGTGTTATCGAT

CAGAATGCTCTGGAGATCATTTTCGCCGCCGGTATACAACAACGGGCCGCGACCAATATG

TTAATCAAACCACTGGTGGTCAGTATCATCAGGCAACGCCCCGTCATGGAATATGATGCA

TCCCATCTCGGCAATATGGTTAACCGACTGGAAGAAGCATTACCCCCGGAGTTACCGGCA

TAA

**>6703|6261647|ETEC_3218A|integral membrane protein [Escherichia coli ETEC H10407]**

ATGTTACAGATAGTCGGCGCGCTGATCCTGCTGATCGCAGGATTTGCCATTCTTCGCCTT

TTGTTCAGAGCATTAATCAGCACGGCTTCTGCGCTGGCAGGGCTCATATTGCTGTGTCTG

TTCGGCCCGGCCTTACTGGCTGGCTATATCACCGAACGCATAACCCGGTTGTTCCATATT

CGCTGGCTGGCAGGCGTATTTCTGACGATTGCCGGAATGATCATCAGCTTCATGTGGGGA

CTTGATGGTAAACATATCGCGCTGGAGGCTCACACCTTTGACTCTGTGAAATTTATTCTG

ACCACCGCTCTCGCCGGTGGTCTGCTGGCTGTTCCCCTGCAGATCAAAAACATTCAGCAG

AACGGGATCACACCAGAAGATATCAGCAAGGAAATTAACGGGTATTACTGCTGTTTTTAT

ACTGCCTTTTTCCTTATGGCGTGTTCTGCATGCGCACCATTGATCGCGTTACAGTACGAT

ATTTCACCGTCACTGATGTGGTGGGGCGGGTTGTTGTACTGGCTGGCTGCATTAGTGACG

CTGCTATGGGCGGCCAGCCAGATCCAGGCGCTGAAAAAACTGACCTGTGCCATCAGCCAG

ACACTGGAAGAACAACCGGTGCTCAACAGTAAATCGTGGCTGACCAGTTTGCAAAACGAT

TACAGCCTTCCTGACTCACTGACGGAGCGCATCTGGCTGACGCTCATTTCTCAACGGATT

TCCCGGGGAGAGCTGAGGGAATTTGAACTGGCAGACGGAAACTGGTTACTGAACAATGCC

TGGTATGAAAGAAACATGGCAGGGTTTAACGAACAGTTGAAAGAGAACCTGTCATTCACA

CCTGATGAACTGAAAACGCTCTTCCGAAACCGCCTGAATTTATCACCGGAAGCGAATGAC

GATTTTCTCGATCGTTGCCTGGACGGCGGTGACTGGTATCCCTTTTCAGAAGGTCGCCGT

TTTGTATCATTCCATCACGTGGATGAGCTTCGTATCTGTGCCTCCTGCGGGCTGACAGAA

GTACATCATGCCCCGGAAAATCATAAGCCGGATCCGGAATGGTACTGCTCCTCTCTTTGT

CGCGAAACAGAAACACTGTGTCAGGAAATTCATGAACGCCCTTACAACAGCTTTATTTCC

GATGCAACGGCGAATGGTCTGATTCTCATGAAACTGCCGGAAACCTGGAGTACAAATGAG

AAAATGTTTGCTTCCGGAGGGCAGGGACATGGGTTTGCCGCTGAACGGGGAAACCATATT

GTCGACAGAGTCCGTCTGAAAAACGCACGGATCCTCGGTGATAATAATGCCAGAAATGGA

GCAGACAGACTGGTCAGCGGAACAGAAATCCAGACGAAATATTGTTCAACTGCAGCCCGT

AGCGTCGGTGCGGCATTCGACGGACAAAACGGACAGTATCGTTACATGGGAAATAATGGC

CCCATGCAACTGGAAGTCCCCCGTGATCAGTATGCCGGCGCTGTTGAAACCATGAGGAAT

AAGATCCGCGAAGGTAAAGTACCCGGTGTAACCGATCCCGCAGAAGCGTCCCGGCTGATT

CGTCGGGGACATCTGACTTATACTCAGGCCCGTAATATCACCCGGTTCGGGACCATCGAA

TCGGTCACTTATGATATTGCCGAGGGTTCGGTTGTCAGTCTGGCGGCCGGAGGGATCAGT

TTTGCCCTGACGGCATCGGTCTTCTGGCTCAGCACCGGCGATCGCGATGCTGCCCTGCAG

ACAGCTGCTGTCCAGGCAGGAAAAACCTTCACCCGCACACTGGCTGTCTACGTCACAACC

CAGCAACTCCACCGGCTCAGTGTTGTTCAGGGTATGCTGAAGCACATTGATTTTTCGACT

GCCAGCCCGACTGTCCGACTGGCTCTTCAGAAGGGGACCGGTGCAGGAAATATCAGTGCC

CTGAACAAAGTGATGAAGGGTACGCTGGTGACATCTCTGGCACTGGTAGCTGTCACAACC

GGCCCTGACATGATCAAAATGTTGCGGGGACGGATCTCCGGTACGCAGTTCATCAGGAAT

CTTGCCGTGGCATCTTCCGGTGTGGCAGGTGGTGCTGTCGGGTCAGTGGCGGGCGGAATA

CTGTTCAGTCCACTGGGACCATTTGGTGCACTGACAGGGCGTGTGGTTGGCGGTGTTCTG

GGGGGAATGATTGCCTCCGCTGTATCAGGAAAAATTGCCGGAGCGCTGGTTGAAGAAGAT

CGCGTCAAAATTCTGGCAATGATTCAGGAGCAGGTGACATGGCTTGCCGGCAGTTTCCTG

CTGACCGGACATGAGATTGAAAATCTGAACGAGAATCTGGCCCGTGTTATCGATCAGAAT

GCTCTGGAGATCATTTTCGCCGCCGGTATACAACAACGGGCCGCGACCAATATGTTAATC

AAACCACTGGTGGTCAGTATCATCAGGCAACGCCCCGTCATGGAATATGAAGCATCCCAT

CTCGGCAATATGGTTAACCGACTGGAAGAAGCATTACCCCCGGAGTTACCGGCATAA

**>6703|6264671|ETEC_4461|putative inner membrane protein [Escherichia coli ETEC H10407]**

GTGACTATGTTACAGATAGTCGGCGCGCTGATCCTGCTGATCGCAGGATTTGCCATTCTT

CGCCTTTTGTTCAGAGCATTAATCAGCACGGCTTCTGCGCTGGCAGGGCTCATATTGCTG

TGTCTGTTCGGCCCGGCCTTACTGGCTGGCTATATCACCGAACGCATAACCAGGTTGTTC

CATATTCGCTGGCTGGCAGGCGTATTTCTGACGATTGCCGGAATGATCATCAGCTTCATG

TGGGGACTTGATGGTAAACATATCGCGCTGGAGGCTCACACCTTTGACTCTGTGAAATTT

ATTCTGACCACCGCTCTCGCCGGTGGTCTGCTGGCTGTTCCCCTGCAGATCAAAAACATT

CAGCAGAACGGGATCACACCAGAAGATATCAGCAAGGAAATTAACGGGTATTACTGCTGT

TTTTATACTGCCTTTTTCCTTATGGCGTGTTCTGCATGCGCACCATTGATCGCGTTACAG

TACGATATTTCACCGTCACTGATGTGGTGGGGCGGGTTGTTGTACTGGCTGGCTGCATTA

GTGACGCTGCTATGGGCGGCCAGCCAAATCCAGGCGCTGAAAAAACTGACCTGTGCCATC

AGCCAGACACTGGAAGAACAACCGGTGCTCAACAGTAAATCGTGGCTGACCAGTTTGCAA

AACGATTACAGCCTTCCTGACTCACTGACGGAGCGCATCTGGCTGACGCTCATTTCTCAA

CGGATTTCCCGGGGAGAGCTGAGGGAATTTGAACTGGCAGACGGAAACTGGTTACTGAAC

AATGCCTGGTATGAAAGAAACATGGCAGGGTTTAACGAACAGTTGAAAGAGAACCTGTCA

TTCACACCTGATGAACTGAAAACGCTCTTCCGAAACCGCCTGAATTTATCACCGGAAGCG

AATGACGATTTTCTCGATCGTTGCCTGGACGGCGGTGACTGGTATCCCTTTTCAGAAGGT

CGCCGTTTTGTATCATTCCATCACGTGGATGAGCTTCGTATCTGTGCCTCCTGCGGGCTG

ACAGAAGTACATCATGCCCCGGAAAATCATAAGCCGGATCCGGAATGGTACTGCTCCTCT

CTTTGTCGCGAAACAGAAACACTGTGTCAGGAAATTCATGAACGCCCTTACAACAGCTTT

ATTTCCGATGCAACGGCGAATGGTCTGATTCTCATGAAACTGCCGGAAACCTGGAGTACA

AATGAGAAAATGTTTGCTTCCGGAGGGCAGGGACATGGGTTTGCCGCTGAACGGGGAAAC

CATATTGTCGACAGAGTCCGTCTGAAAAACGCACGGATCCTCGGTGATAATAATGCCAGA

AATGGAGCAGACAGACTGGTCAGCGGAACAGAAATCCAGACGAAATATTGTTCAACTGCA

GCCCGTAGCGTCGGTGCGGCATTCGACGGACAAAACGGACAGTATCGTTACATGGGAAAT

AATGGCCCCATGCAACTGGAAGTCCCCCGTGATCAGTATGCCGGCGCTGTTGAAACCATG

AGGAATAAGATCCGCGAAGGTAAAGTACCCGGTGTAACCGATCCCGCAGAAGCGTCCCGG

CTGATTCGTCGGGGACATCTGACTTATACTCAGGCCCGTAATATCACCCGGTTCGGGACC

ATCGAATCGGTCACTTATGATATTGCCGAGGGTTCGGTTGTCAGTCTGGCGGCCGGAGGG

ATCAGTTTTGCCCTGACGGCATCGGTCTTCTGGCTCAGCACCGGCGATCGCGATGCTGCC

CTGCAGACAGCTGCTGTCCAGGCAGGAAAAACCTTCACCCGCACACTGGCTGTCTACGTC

ACAACCCAGCAACTCCACCGGCTCAGTGTTGTTCAGGGTATGCTGAAGCACATTGATTTT

TCGACTGCCAGCCCGACTGTCCGACTGGCTCTTCAGAAGGGGACCGGTGCAGGAAATATC

AGTGCCCTGAACAAAGTGATGAAGGGTACGCTGGTGACATCTCTGGCACTGGTAGCTGTC

ACAACCGGCCCTGACATGATCAAAATGTTGCGGGGACGGATCTCCGGTACGCAGTTCATC

AGGAATCTTGCCGTGGCATCTTCCGGTGTGGCAGGTGGTGCTGTCGGGTCAGTGGCGGGC

GGAATACTGTTCAGTCCACTGGGACCATTTGGTGCACTGACAGGGCGTGTGGTTGGCGGT

GTTCTGGGGGGAATGATTGCCTCCGCTGTATCAGGAAAAATTGCCGGAGCGCTGGTTGAA

GAAGATCGCGTCAAAATTCTGGCAATGATTCAGGAGCAGGTGACATGGCTTGCCGGCAGT

TTCCTGCTGACCGGACATGAGATTGAAAATCTGAACGAGAATCTGGCCCGTGTTATCGAT

CAGAATGCTCTGGAGATCATTTTCGCCGCCGGTATACAACAACGGGCCGCGACCAATATG

TTAATCAAACCACTGGTGGTCAGTATCATCAGGCAACGCCCCGTCATGGAATATGAAGCA

TCCCATCTCGGCAATATGGTTAACCGACTGGAAGAAGCATTACCCCCGGAGTTACCGGCA

TAA

**>6703|7968233|EO104H4LBv1_920021|conserved hypothetical protein; putative membrane protein, yeeR [Escherichia coli O104:H4 LB226692]**

ATGTTACAGATAGTCGGCGCGCTGATTCTGCTGATCGCAGGATTTGCCATTCTTCGCCTT

TTGTTCAGAGCATTAACCAGCACAGCGTCTGCGCTGGCAGGGTTCATATTGCTGTGTCTG

TTCGGTCCGGCTTTACTGGCTGGCTATATCACTGAACGCATAACCCGGTTATTCCATATT

CGCTGGCTGGCAGGCGTATTTCTGACGATTGCCGGAATGATCATCAGCTTCATGTGGGGA

CTTGATGGTAAACATATCGCACTGGAGGCTCATACTTTTGACTCTGTAAAATTTATTCTG

ACCACCGCTCTCGCCGCTGGTCTGCTGGCTCTTCCCGTGCAGATAAGAACCATTCAGCAG

AACGGGCTCACACCTGAAGATATCAGCAAGGAAATTAACGGGTATTACTGCTGTTTTTAT

ACTGCCTTTTTCCTTATGGCGTGTTCTGCATACGCACCATTGATCGCATTGCAGTTCGAT

ATTTCACCCTCACTGATGTGGTGGGGCGGGTTGTTGTACTGGCTGGCTGCATTAGTGACG

CTGCTATGGGCGGCCAGCCAGATCCAGGCGCTGAAAAGACTGACCAGTGCCATCAGCCAG

ACACTGGAAGAACAACCGGTGCTCAACAGTAAATCATGGCTGAGCAGTTTGCAAAACGAT

TACAGCCTTCCTGAAACGCTGACGGAGCGCATCTGGCTGACACTCATTTCACAACGGATT

TCCCGGGGAGAACTGAGGGAATTTGAACTGGCAGACGGAAACTGGCTACTGGACAATGCC

TGGTATGAAAGAAACATGGCGGGTTTCAACGAAAAGCTGAGAGAGAGCCTGTCATTTACC

CCTGATGAACTGAAAACCCTCTTCCGGAACCGCCTGAATTTATCACCGGAAGCGAATGAC

GATTTTCTCGATCGTTGCCTGGACGGCGGTGACTGGTATCCCTTTTCAGAAGGTCGCCGT

TTTGTATCATTCCATCACGTGGATGAGCTTCGTATCTGTGCCTCCTGCGGGCTGACAGAA

GTACATCATGCCCCGGAAAATCATAAGCCGGATCCGGAATGGTACTGCTCCTCTCTTTGT

CGCGAAACAGAAACACTGTGTCAGGAAATTTATGAACGCCCCTACAACAGTTTTATTTCC

GATGCAACGGCGAATGGTCTGATTCTCATGAAACTGCCGGAAACCTGGAGTACAAATGAG

AAAATGTTTGCTTCCGGAGGGCAGGGACATGGGTTTGCCGCTGAACGGGGAAACCATATT

GTCGACAGAGTCCGTCTGAAAAACGCACGGATCCTCGGTGATAATAATGCCAGAAATGGA

GCAGACAGACTGGTCAGCGGAACAGAGATCCAGACGAAATATTGTTCAACTGCAGCCCGT

AGCGTCGGTGCGGCATTCGACGGACAAAACGGACAGTATCGTTACATGGGAAATCATGGC

CCCATGCAACTGGAAGTCCCCCGTGATCAGTATGCCGGCGCTGTTGAAACCATGAGGAAT

AAGATCCGCGAAGGTAAAGTACCTGGTGTAACCGATCCCGCAGAAGCGTCCCGGCTGATT

CGTCGGGGACATCTGACTTATACCCAGGCCCGTAATATCACCCGGTTCGGGACCATCGAA

TCGGTCACTTATGATATTGCCGAGGGTTCGGTTGTCAGTCTGGCGGCCGGAGGGATCAGT

TTTGCCCTGACGGCATCGGTCTTCTGGCTCAGCACCGGCGATCGCGATGCTGCCCTGCAG

ACAGCTGCTGTCCAGGCAGGAAAAACCTTCACCCGCACACTGGCTGTCTACGTCACAACC

CAGCAACTTCACCGGCTCAGTGTTGTTCAGGGTATGCTGAAGCACATTGATTTTTCGACG

GCCAGTCCGACTGTCCGACTGGCTCTTCAGAAGGGGACCGGTACAGGAAATATCAGTGCC

CTGAACAAAGTGATGAAGGGTACGCTGGTGACATCTCTGGCACTGGTAGCTGTCACAACC

GGCCCTGACATGATCAAAATGTTGCGGGGACGGATCTCCGGTACGCAGTTCATCAGGAAT

CTTGCCGTGGCATCTTCCGGTGTGGCAGGTGGAGCTGTCGGGTCAGTGGCGGGCGGGATA

CTGTTCAGTCCACTGGGACCATTTGGTGCACTGACAGGGCGTGTGGTTGGCGGTGTTCTG

GGGGGAATGATTGCCTCCGCTGTATCAGGAAAAATTGCCGGAGCGCTGGTTGAAGAAGAT

CGCGTCAAAATTCTGGCAATGATTCAGGAACAGGTGACATGGCTTGCCGGCAGTTTCCTG

CTGACCGGACATGAAACAGAAAATCTGAACGCGAATCTGGCCCGTGTTATCGATCAGAAT

GCTCTGGAGATCATTTTCGCCGCCGGTATACAACAACGGGCCGCGACCAATATGTTAATC

AAACCACTGGTGGTCAGTATCATCAGGCAACGCCCCGTCATGGAATATGATGCATCCCAT

CTCGGCAATATGGTTAACCGGCTGGAAGAAGCATTACCCCCGGAGTTACCGGCATAA

**>6703|29771411|ECNA114_4689|hypothetical protein [Escherichia coli NA114]**

ATGAAGAATAAGATCCGCGAAGGTAAAGTACCCGGTGTAACCGATCCCGCAGAAGCGTCC

CGGCTGATTCGTCGGGGACATCTGACTTATACCCAGGCCCGTAATATCACCCGGTTCGGG

ACCATCGAATCGGTCACTTATGATATTGCCGAGGGGTCGGTTGTCAGTCTGGCGGCCGGA

GGGATCAGTTTTGCCCTGACGGCATCGGTCTTCTGGCTCAGCACCGGCGATCGCGATGCT

GCCCTGCAGACAGCTGCTGTCCAGGCAGGAAAAACCTTCACCCGCACACTGGCTGTCTAC

GTCACAACCCAGCAACTTCACCGGCTCAGTGTTGTTCAGGGTATGCTGAAGCATATTGAT

TTTTCGACGGCCAGCCCGACTGTCCGGCAGGCGCTTCAGAAGGGGACCGGTGCAGGAAAT

ATCAGTGCCCTGAACAAAGTGATGAAGGGTTCGCTGGTGACATCTCTGGCACTGGTAGCT

GTCACAACCGGCCCTGACATGATCAAAATGTTGCGGGGACGGATCTCCGGTGCGCAGTTC

ATCAGGAATCTTGCCGTGGCATCTTCCTGTGTGGCAGGTGGTGCTGTCGGGTCAGTGGCG

GGCGGGATATTGTTCAGTCCACTGGGACCATTTGGTGCACTGACAGGGCGTGTGGTTGGC

GGTGTTCTGGGGGGAATGATTGCCTCCGCTGTATCAGGAAAAATTGCCGGAGCGCTGGTT

GAAGAAGATCGCGTCAAAATTCTGGCAATGATTCAGGAGCAGATGACATGGCTTGCCGGC

AGTTTCCTGCTGACCGGACATGAGATTGAAAATCTGAACGCGAATCTGGCCCGTGTTATC

GATCAGAATGCTCTGGAGATCATTTTCGCCGCCGGTATACAACAACGGGCCGCGACCAAC

ATGTTAATCAAACCACTGGTGGTCAGTATCATCAGGCAACGCCCCGTCATGGAATATGAT

GCATCCCATCTCGGCAATATGGTTAACCGACTGGAAGAAACATTACCCCCGGAGTTACCG

GCATAA

**>7708|3055750|ECUMN_3402|conserved hypothetical protein; putative exported protein similar to Aec69 [Escherichia coli UMN026]**

ATGATTCACCTGTTCAAAACCTGCATGATTACCGCCTTCATTCTGGGGTTAACGTGGTCT

GCCCCACTCCGGGCACAGGATCAACGTTACATCAGTATACGCAATACAGATACGATATGG

CTCCCGGGAAATATTTGTGCTTACCAGTTCCGGCTGGATAATGGCGGAAACGATGAAGGA

TTTGGCCCCCTCACCATCACTCTGCAACTCAAAGACAAATATGCTCAGACGCTGGTGACC

AGAAAAATGGAAACGGAAGCCTTTGGTGACAGTAATGCCACGCGAACCACAGACGCATTT

CTGGAAACGGAGTGCGTGGAAAATGTCGCCACAACCGAAATCATTAAAGCAACTGAAGAA

AGTAACGGCCATCGTGTCAGTCTGCCGTTATCGGTTTTCGATCCCCAGGACTACCATCCA

CTGCTGATTACCGTTTCCGGAAAAAATGTTAACTGA

**>7708|938001|EC42_4698|conserved hypothetical protein; putative exported protein similar to Aec69 [Escherichia coli 042]**

ATGATTCACCTGTTCAAAACCTGCATGATTACCGCCTTCATTCTGGGGTTAACGTGGTCT

GCCCCACTCCGGGCACAGGATCAACGTTACATCAGTATACGCAATACAGATACGATATGG

CTCCCGGGAAATATTTGTGCTTACCAGTTCCGGCTGGATAATGGCGGAAACGATGAAGGG

TTTGGCCCCCTCACCATCACTCTGCAACTCAAAGACAAATATGGTCAGACGCTGGTGACC

AGAAAAATGGAAACGGAAGCTTTTGGTGACAGTAATGCCACGCGAACCACAGACGCATTT

CTGGAAACGGAGTGCGTGGAAAATGTCGCCACAACCGAAATCATTAAAGCAACTGAAGAA

AGTAACGGCCATCGTGTCAGTCTGCCGTTATCGGTTTTCGATCCCCAGGACTACCATCCA

CTGCTAATTACCGTTTCCGGAAAAAACGTTAACTGA

**>7708|935627|EC42_2324|conserved hypothetical protein, similar to Aec69 [Escherichia coli 042]**

ATGATTCACCTGTTCAAAACCTGCATGATTACCGCCTTCATTCTGGGGTTAACGTGGTCT

GCCCCACTCCGGGCACAGGATCAACGTTACATCAGTATACGCAATACAGATACGATATGG

CTCCCGGGAAATATTTGTGCTTACCAGTTCCGGCTGGATAATGGCGGAAACGATGAAGGA

TTTGGCCCCCTCACCATCACTCTGCAACTCAAAGACAAATATGGTCAGACGCTGGTGACC

AGAAAAATGGAAACGGAAGCTTTTGGTGACAGTAATGCCACGCGAACCACAGACGCATTT

CTGGAAACGGAGTGCGTGGAAAATGTCGCCACAACCGAAATCATTAAAGCAACTGAAGAA

AGTAACGGCCATCGTGTCAGTCTGCCGTTATCGGTTTTCAATCCCCAGGACTACCATCCA

CTGCTGATTACCGTTTCCGGAAAAAACGTTAACTGA

**>7708|6261648|ETEC_3219|putative exported protein [Escherichia coli ETEC H10407]**

ATGATTCACCTGTTCAAAACCTGCATGATTACCACCTTCATTCTGGGGTTAATGTGGTCT

GCCCCACTCCGGGCACAGGATCAACGTTACATCAGTATACGCAATACAGATACGATATGG

CTCCCGGGAAATATTTGTGCTTACCAGTTCCGGCTGGATAATGGCGGAAACGATGAAGGA

TTTGGCCCCCTCACCATCACTCTGCAACTCAAAGACAAATATGGTCAGACGCTGGTGACC

AGAAAAATGGAAACGGAAGCTTTTGGTGACAGTAATGCCACGCGAACCACAGACGCATTT

CTGGAAACGGAGTGCGTGGAAAATGTCGCCACAACCGAAATCATCAAAGCAACTGAAGAA

AGTAACGGCCATCGTGTCAGTCTGCCGTTATCGGTTTTCAATCCCCAGGACTACCATCCA

CTGCTGATTACCGTTTCCGGAAAAAACGTTAACTGA

**>7708|6264670|ETEC_4460|conserved hypothetical protein [Escherichia coli ETEC H10407]**

ATGATTCACCTGTTCAAAACCTGCATGATTACCACCTTCATTCTGGGGTTAATGTGGTCT

GCCCCACTCCGGGCACAGGATCAACGTTACATCAGTATACGCAATACAGATACGATATGG

CTCCCGGGAAATATTTGTGCTTACCAGTTCCGGCTGGATAATGGCGGAAACGATGAAGGA

TTTGGCCCCCTCACCATCACTCTGCAACTCAAAGACAAATATGGTCAGACGCTGGTGACC

AGAAAAATGGAAACGGAAGCTTTTGGTGACAGTAATGCCACGCGAACCACAGACGCATTT

CTGGAAACGGAGTGCGTGGAAAATGTCGCCACAACCGAAATCATTAAAGCAACTGAAGAA

AGTAACGGCCATCGTGTCAGTCTGCCGTTATCGGTTTTCGATCCCCAGGACTACCATCCA

CTGCTGATTACCGTTTCCGGAAAAAACGTTAACTGA

**>7708|7968234|EO104H4LBv1_920022|conserved hypothetical protein; putative exported protein similar to Aec69 [Escherichia coli O104:H4 LB226692]**

ATGATTCACCTGTTCAAAACCTGCATGATTACCGCCTTCATTCTGGGGTTAACGTGGTCT

GCCCCACTCCGGGCACAGGATCAACGTTACATCAGTATACGCAATACAGATACGATATGG

CTCCCGGGAAATATTTGTGCTTACCAGTTCCGGCTGGATAATGGCGGAAACGATGAAGGG

TTTGGCCCCCTCACCATCACTCTGCAACTCAAAGACAAATATGGTCAGACGCTGGTGACC

AGAAAAATGGAAACGGAAGCTTTTGGTGACAGTAATGCCACGCGAACCACAGACGCATTT

CTGGAAACGGAGTGCGTGGAAAATGTCGCCACAACCGAAATCATTAAAGCAACTGAAGAA

AGTAACGGCCATCGTGTCAGTCTGCCGTTATCGGTTTTCGATCCCCAGGACTACCATCCA

CTGCTAATTACCGTTTCCGGAAAAAACGTTAACTGA

**>7708|29771412|ECNA114_4690|hypothetical protein [Escherichia coli NA114]**

ATGATTCACCTGTTCAAAACCTGCATGATTACCGCCTTCATTCTGGGGTTAACGTGGTCT

GCCCCACTCCGGGCACAGGATCAACGTTACATCAGTATACGCAATACAGATACGATATGG

CTCCCGGGAAATATTTGTGCTTACCAGTTCCGGCTGGATAATGGCGGAAACGATGAAGGA

TTTGGCCCCCTCACCATCACTCTGCAACTCAAAGACAAATATGGTCAGACGCTGGTGACC

AGAAAAATGGAAACGGAAGCCTTTGGTGACAGTAATGCCACGCGAACCACAGACGCATTT

CTGGAAACGGAGTGCGTGGAAAATGTCGCCACAACCGAAATCATTAAAGCAACTGAAGAA

AGTAACGGCCATCGTGTCAGTCTGCCGTTATCGGTTTTCGATCCCCAGGACTACCATCCA

CTGCTGATTACCGTTTCCGGAAAAAACGTTAACTGA

**>17545|3055741|ECUMN_3394|fragment of hemolysin expression-modulating protein [Escherichia coli UMN026]**

GTGATTTCCCACACACGTTATAAACTTACCCCTGCGGAGCTGGAAGCCTTTAACTCTGCG

GTCGATCACCGCCTGGCAGAACTGACAATGAACAAACTTTACGATCGTGTGCCGGCTTCC

GTCTGGAAATATGTCACCTGA

**>17545|938009|EC42_4706|haemolysin expression-modulating protein [Escherichia coli 042]**

ATGGAAGTTAAAACCAAAGAAGACTGGCTGTATCAGTTCCGTCGTTGTTCATCCCGGGAG

ACGCTGGAAAAAGTGATTTCCCACACACGTTATAAACTTACCCTTGCGGAGCTGGAAGCC

TTCAACTCTGCGGTTGATCACCGACTGGCAGAACTAACCATGAACAAACTTTACGATCGC

GTTCCGGCTTCTGTCTGGAAATATGTTACCTGA

**>17545|935620|EC42_2317|fragment of putative transcriptional regulator (partial) [Escherichia coli 042]**

GTGATTTCCCACACGCGTTATAAACTTACCTCTGCGGAACTGGAAGCCTTCAACTCTGTG

GTTGATCACTGGCTGGCAGAACTGACAATGAACAAGCTTTACGATCGTGTTCCCGCTTCA

GTCTGGAAATATGTCATCTGA

**>17545|938317|EC42_5014|putative transcriptional regulator [Escherichia coli 042]**

GTGGCAGCCCGTAATACGTCAGGAGACGCGGGAAAAGTGAATTCCCACACGCGTTATAAA

CTTACCCTTGCGGAGCTGGAAGCCTTTAACTCTGCCGTTGACAACCGGCTGGCAGAACTG

ACAATGAACAAACTTTACGATCGCGCGCCGGCTTCCGTCTGGAAATATGTCACCTGA

**>17545|6259866|ECOET_3441|putative hemolysin expression modulating protein [Escherichia coli ETEC H10407]**

GTGATTTCCCATACGCGTTATAAACTTACCCCTGCGGAGATGGAAGCCTTCAACTCTGCG

GTCGATCACCGACTGGCAGAACTGACAATGAACAAGCTTTACGATCGTGTTCCCGCTTCA

GTCTGGAAATATGTCATCTGA

**>17545|6263383|ETEC_2114|putative regulatory protein [Escherichia coli ETEC H10407]**

GTGGCAGCCCGTAATACGTCAGGAGACGCGGGAAAAGTGAATTCCCACACGCGTTATAAA

CTTACCCTTGCGGAGCTGGAAGCCTTTAACTCTGCCGTTGACAACCGGCTGGCAGAACTG

ACAATGAACAAACTTTACGATCGCGCGCCGGCTTCCGTCTGGAAATATGTCACCTGA

**>17545|7968224|EO104H4LBv1_920012|haemolysin expression-modulating protein [Escherichia coli O104:H4 LB226692]**

ATGGAAGTTAAAACCAAAGAAGACTGGCTGTATCAGTTCCGTCGTTGTTCATCCCGGGAG

ACGCTGGAAAAAGTGATTTCCCACACACGTTATAAACTTACCCTTGCGGAGCTGGAAGCC

TTCAACTCTGCGGTTGATCACCGACTGGCAGAACTAACCATGAACAAACTTTACGATCGC

GTTCCGGCTTCTGTCTGGAAATATGTTACCTGA

**>17545|7969845|EO104H4LBv1_1090077|putative transcriptional regulator [Escherichia coli O104:H4 LB226692]**

GTGGCAGCCCGTAATACGTCAGGAGACGCGGGAAAAGTGAATTCCCACACGCGTTATAAA

CTTACCCTTGCGGAGCTGGAAGCCTTTAACTCTGCCGTTGACAACCGGCTGGCAGAACTG

ACAATGAACAAACTTTACGATCGCGCGCCGGCTTCCGTCTGGAAATATGTCACCTGA

**>17545|7970066|EO104H4LBv1_1210003|Modulating protein ymoA [Escherichia coli O104:H4 LB226692]**

ATGGCAAAAACAAAACAGGAATGGTTATATCAGTTACGTCGCTGTTCCAGCTTAAAGACA

CTTGAGAAAATTATTGCTAAAAACCAGGGCACTTTAGCCTCGGATAAAATAGAAGCATTC

AACTCTGCAGTTGATCATCGCCTTGCGGAACTGACAATGAACAAATTGTACGACAAAGTA

CCGGCTTCTGTCTGGAAGCATGTAAAATAA

**>17545|7970627|EO104H4LBv1_3420001|putative transcriptional regulator [Escherichia coli O104:H4 LB226692]**

TTGTTCATCCCGGGAGACACTGGAAAAGTGATTTCCCACACGCGTTATAAACTTACCCCT

GCGGAGCTGGAAACCTTCAACTCTGCGGTCGATCACCGACTGGCAGAACTGACAATGAAC

AAGCTTTACGATCGTGTTCCCGCTTCAGTCTGGAAATATGTCATCTGA

**>17545|29766537|ECNA114AM_2013|modulator of gene expression, with H-NS (fragment) [Escherichia coli NA114]**

GTGATTTCCCACACACGTTATAAACTTACCCCTGCGGAGCTGGAAGCCTTTAACTCTGCC

GTTGACAACCGGCTGGCAGAACTGACAATGAACAAACTTTACGATCGCGTGCCGGCTTCC

GTCTGGAAATATGTCACCTGA
